# Modelling the cost-effectiveness of interventions to treat or prevent neuropathic ulcers arising from leprosy: application to L-PRF

**DOI:** 10.1186/s12962-026-00739-8

**Published:** 2026-03-26

**Authors:** Jessica Ochalek, Naomi Kate Gibbs, Rita Faria, Indra Bahadur Napit, Dilip Shrestha, Karuna Neupane, Anju Adhikari, Joydeepa Darlong, Karthikeyen Govindasamy, Richard Lilford, Pedro Saramago, Mark Sculpher

**Affiliations:** 1 Centre for Health Economics, University of York, York, UK; 2 Anandaban Hospital, The Leprosy Mission Nepal, Lalitpur, Nepal; 3 Research Domain, The Leprosy Mission Trust India, New Delhi, India; 4 Warwick Centre for Global Health, Warwick Medical School, University of Warwick, Coventry, CV4 7AL UK; 5 Institute of Applied Health Research, University of Birmingham, Birmingham, UK

**Keywords:** Cost-effectiveness, Decision-modelling, Decision-making, Opportunity cost, Leprosy, Ulcer

## Abstract

**Background:**

Leprosy remains a public health problem in low- and middle-income countries (LMIC), often causing extensive nerve damage leading to neuropathic ulcers with significant morbidity. Whether interventions to prevent and treat such ulcers offer good value for money requires modelling their lifetime costs and benefits and consideration of opportunity costs. This paper provides the first decision analytical model for the economic evaluation of interventions to manage neuropathic ulcers in leprosy, and applies it to assess the cost-effectiveness of leukocyte- and platelet-rich fibrin (L-PRF) ulcer dressings in Nepal.

**Methods:**

A discrete time Markov model is developed in consultation with clinical experts and informed by reviews of the literature. It is parameterised using evidence from the literature and from a trial of L-PRF enabling the model to be adapted to evaluate a range of interventions. To estimate the effectiveness of L-PRF, we implement parametric survival modelling to extrapolate time to healing beyond the trial follow-up period and calculate the relative risk of recurrence. Costs and effects are assessed over patients’ lifetimes.

**Results:**

No existing model of neuropathic ulcers arising from leprosy in LMIC was identified in the literature, and literature to inform model parameters is scarce. Our model-based analysis demonstrates how models can be parameterised, including from trial data. Our application of the model found that L-PRF increased mean costs by 6,731 NRP per patient and generated 0.006 additional QALYs compared with saline dressings. At an estimated health opportunity cost threshold of 38,970 NRP per QALY, the incremental net health benefit was − 0.167 QALYs per patient (− 167 per 1,000 patients), with substantial uncertainty. Under these assumptions, L-PRF was unlikely to be cost-effective in the hospital setting analysed. Given uncertainty in the data, there is potentially significant value from generating additional trial evidence.

**Conclusion:**

This study presents a structured decision-analytic framework for evaluating neuropathic ulcer interventions in leprosy, informed by limited available data. Researchers intending to use the model to evaluate interventions should carefully consider whether the parameters estimated here are relevant to their clinical and geographical context and update these as required.

**Trial registration:**

ClinicalTrials.gov ISRCTN14933421. Registered on 16 June 2020.

**Supplementary Information:**

The online version contains supplementary material available at 10.1186/s12962-026-00739-8.

## Background

Leprosy, a chronic infectious disease caused by *Mycobacterium leprae*, continues to pose significant public health challenges, particularly in low- and middle-income countries (LMIC). Despite extensive global efforts to control and eliminate the disease, leprosy remains endemic in several regions, with thousands of new cases reported annually [1, 2].

Classified by the World Health Organization (WHO) as a neglected tropical disease (NTD), leprosy like most NTDs, is strongly associated with poverty and socio-economic disadvantage, both within and across countries [3]. The global burden is highly concentrated: in 2019, 79% of new cases were reported in India, Brazil and Indonesia, all LMIC [4].

One of the most common complications of leprosy is the development of neuropathic ulcers, which result from the extensive nerve damage characteristic of the disease. These ulcers lead to significant morbidity and disability [5]. Effective management of leprosy ulcers is crucial for improving patient outcomes and health-related quality of life. Traditional wound care practices, including regular cleaning, debridement, and the use of protective footwear, are essential but often inadequate in promoting rapid and complete healing [6, 7]. The introduction of advanced wound healing treatments offers the potential for improved healing rates and reduced recurrence, which could translate into significant cost savings over time [8]. However, the economic implications of these advanced treatments need thorough evaluation to guide healthcare policy and resource.

Assessing whether interventions to treat or prevent leprosy ulcers offer good value for money requires modelling the lifetime costs and benefits resulting from these interventions, and consideration of the opportunity cost of funding the intervention. The intervention’s opportunity cost is the health that could have been gained by patients if the money required to fund the intervention was instead spent on other healthcare. While well-designed randomised controlled trials can inform the effectiveness of new treatments, a decision model enables the incorporation of evidence from multiple trials or sources to be synthesized, extrapolation of evidence over the appropriate time horizon, generalisation of results to settings and patient groups beyond a trial, and is able to indicate the need for and potential value of further research [9].

Through a systematic literature review, we identified a gap in the literature around decision model-based economic evaluations of interventions for the prevention and treatment of leprosy ulcers in LMIC. The absence of decision models in this area represents a significant gap: decision models are particularly important for chronic and recurrent conditions like leprosy ulcers, where the disease process may involve multiple stages and risks over the patients’ lifetime. So that the decision model can capture the consequences of health conditions and the impact of health interventions, its structure should capture the natural history of the condition and how interventions affect it.

The natural history of leprosy ulcers involves a cycle of ulcer formation, healing, recurrence, and, in some cases, progression to a complicated ulcer [7, 10]. Ulcers typically develop due to neuropathy-related injuries that go unnoticed [6, 7], leading to prolonged healing times and increased risk of infection. Once healed, ulcers may recur, particularly if underlying causes—such as neuropathy or poor protective practices—remain unaddressed. Ulcers can reduce the health-related quality of life of patients and result in healthcare system costs [11, 12].

The model developed here provides a structured framework for assessing the long-term costs and health outcomes of various leprosy ulcer wound treatment and prevention interventions, which can inform resource allocation in LMIC settings. A targeted literature review was conducted to inform transition probabilities between states and health state utilities. To demonstrate how the model can be used to determine the value of interventions for leprosy ulcers in resource-limited settings where leprosy is prevalent, we apply it to assess the cost-effectiveness of Leukocyte-Platelet Rich Fibrin (L-PRF), a novel intervention that aims to improve wound healing time and prevent recurrence [13], in Nepal.

This paper fills the gap in the literature by providing a decision analytical state transition model for the economic evaluation of interventions to manage neuropathic ulcers and demonstrates how the model can be applied by assessing the cost-effectiveness of a novel intervention to improve the speed of ulcer healing and prevent recurrence.

**Fig. 1:**
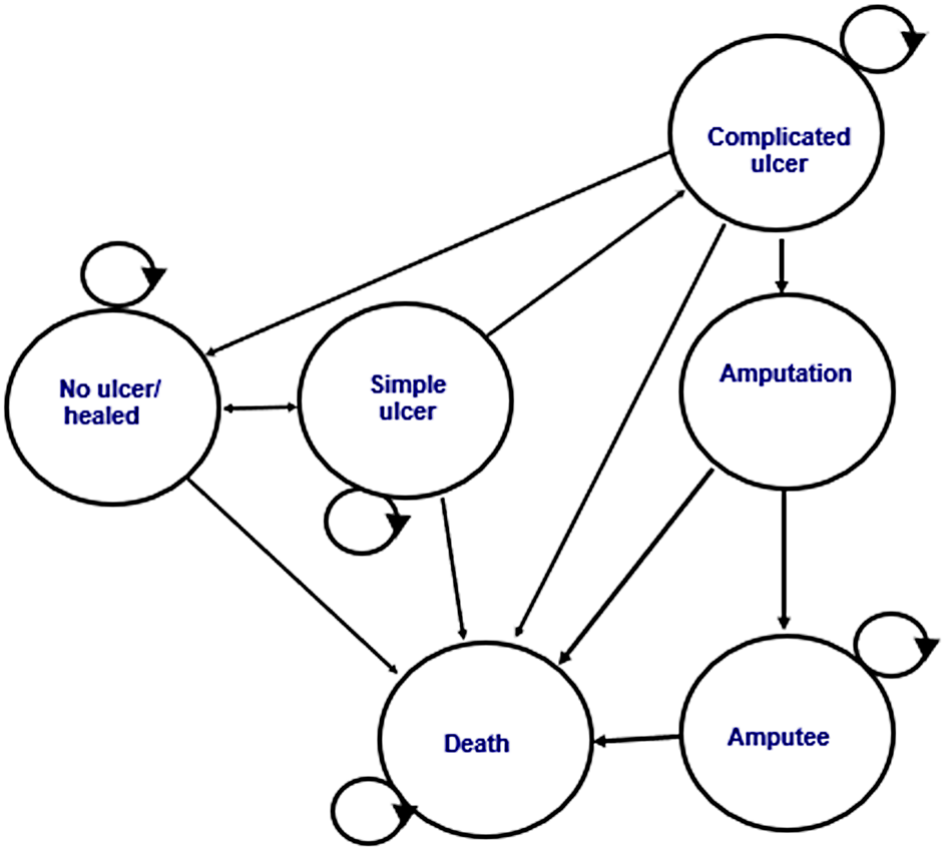
Structure of the Markov state-transition model for the natural history of leprosy-related plantar ulcers

## Methods

### Model structure

We conducted a systematic literature review to inform the development of the model. It covered published model-based economic evaluations pertaining to the treatment and prevention of leprosy ulcers, diabetic foot ulcers (DFU) and Buruli ulcers in LMIC. Models to assess DFU and Buruli ulcers were included because these ulcers have similar consequences to leprosy ulcers despite their different causes [14]. The review was restricted to studies conducted in LMIC to ensure the applicability of results for use in countries where the burden of leprosy is greatest. The methods quality of each paper was assessed using the Philips checklist [15]. Details are provided in Appendix A.

Based on the literature and input from clinical collaborators, the model includes six mutually exclusive health states: no ulcer, simple ulcer, complicated ulcer, amputation, amputee and death. (See Fig. 1). Time is modelled in weekly cycles to represent the chronic nature of ulcer healing and recurrence. The model enables analyses where patients start in the no ulcer/healed model state, the simple ulcer model state or the complicated ulcer model state. In the analysis of L-PRF all patients start in the simple ulcer state. Although the model structure permits patients to enter in the no ulcer state, in the present application this state represents a healed interval among individuals with established neuropathic feet and ongoing recurrence risk, rather than an ulcer-naïve population. Transition probabilities from this state therefore reflect ulcer recurrence rather than first-ulcer incidence.

Neuropathic ulcers exist along a continuum of severity. For modelling purposes, the complicated ulcer state was defined as ulcers involving deep tissue or bone extension (including osteomyelitis), typically requiring surgical intervention (amputation or other surgery). Infections may occur in both simple and complicated ulcers, and patients in either state may incur antibiotic and/or debridement costs. Infections may be present in simple or in complicated ulcers, and patients in either state may therefore incur antibiotic costs and/or debridement (removal of dead or infected tissue). The presence of infection alone was not used to define a separate structural health state, as superficial infection does not necessarily represent structural progression or altered long-term prognosis.

Patients whose complicated ulcers necessitate amputation move to the amputation and then amputee state, where they remain until death since an ulcer cannot recur on the same site following amputation. The amputee state is an absorbing state, reflecting the fact that an ulcer cannot recur when the part of the body on which it occurred was amputated. The amputation state is comprised of four tunnel states (week 1 during which the amputation surgery occurs, and three weeks of healing) enabling the model to account for different probabilities of death and costs in each week. Patients whose complicated ulcers require other types of surgery, such as calcaneus paring, osteotomy, and sequestrectomy, are healed following surgery and so move to the no ulcer/healed state. Following surgery, there is a risk of recurrence of an ulcer on the same site. The probability of recurrence may differ from the probability of a first ulcer occurring on a site. While the probability of recurrence may differ depending on whether a patient has returned to the no ulcer state from the simple or complicated state, in the current model structure, all healed patients are subject to a common recurrence probability. The model does not differentiate recurrence risk by prior ulcer severity or treatment pathway, reflecting limited empirical evidence and the absence of state-memory in the cohort Markov framework.

### Setting

We apply the model to assess the cost-effectiveness of L-PRF in Nepal. In 2023, 2,522 people were diagnosed with leprosy in Nepal, approximately 8.4 per 100,000 population [16]. The Nepali government funds some leprosy-related healthcare through its National Leprosy Eradication Program (NLEP). This includes free multidrug therapy (MDT), which is provided by the World Health Organization (WHO) for all leprosy patients worldwide, and basic healthcare services at government health facilities. The government’s involvement focuses on diagnosis, primary treatment, and follow-up care through health posts and district hospitals. A non-governmental organization, authors’ institute Nepal, often covers additional or specialized care costs and fills gaps in services, including diagnosis, treatment, reconstructive surgeries, rehabilitation, and community support programs. It also funds care for complex cases at its hospitals, such as the authors’ institute, and supports outreach programs in underserved areas.

### Analytical perspective

As life expectancy in Nepal is 70 years, infection with leprosy most commonly occurs in adulthood and leprosy ulcers can recur over people’s lifetime, we use a 60-year time horizon to ensure all costs and benefits are captured. The analysis takes a healthcare system perspective, reflecting the fact that the decision-makers who the analysis aims to inform are authors’ institute Nepal and the Nepal government. While inpatient treatment with L-PRF potentially impacts patients’ productivity and direct non-medical costs, such as the cost of transportation to reach the hospital, only direct medical costs are funded by the authors’ institute Nepal, therefore only inpatient costs are included. Health benefits are measured in terms of quality-adjusted life years (QALYs), which reflect any effects of the intervention on mortality as well as health-related quality of life. Costs and health benefits are discounted at 3% per annum following guidance from WHO and the iDSI Reference Case for health economic evaluation in LMICs [17, 18].

### Intervention and trial of autologous blood products, leukocyte and platelet-rich fibrin (L-PRF), to promote ulcer healing in LEprosy (TABLE) trial

Leukocyte-Platelet Rich Fibrin (L-PRF) is a ‘second generation’ cellular therapy with the potential to improve the healing of chronic ulcers and reduce the risk of complications [19, 20]. It is an advanced autologous biomaterial derived from the patient’s blood through centrifugation. It forms a fibrin matrix rich in platelets, leukocytes, and growth factors, which promote tissue regeneration and wound healing. L-PRF is expected to improve the time to ulcer healing by accelerating tissue repair and to reduce the risk of recurrence by enhancing the quality of wound healing [13, 21].

L-PRF was evaluated in the TABLE trial, in Nepal. Patients were eligible if they were ≥ 18 years of age, had a plantar ulcer of at least six weeks duration, which was not infected and had an area of 2 to 20 cm^2^. The aim was that patients in the trial reflected the population of patients referred from all parts of Nepal to authors’ institute for the treatment of chronic neuropathic ulcers of the foot due to leprosy.

The TABLE trial provides evidence to inform the effectiveness of L-PRF in reducing time to healing and recurrence as well as additional parameters. It was a 1:1 individually randomised trial including 130 participants in which L-PRF was compared to usual care of normal saline dressings where both were applied twice each week [20].

Time to complete healing was measured by both clinicians at dressing changes (i.e., local unblinded) and by blind assessment of ulcer images (i.e., separate to dressing changes and randomised). Dressings were changed twice each week until the clinician deemed the ulcer healed, and again at 6 months post-randomisation. The primary outcomes for the trial were assessed at 42 days, although patients were followed up for 70 days (or until their ulcer healed, whichever was first). After 42 days, patients were allowed to cross-over from control to treatment if their ulcer was determined to be healing slowly. Seven patients (5.4%) switched from saline to L-PRF. Four patients (3.1%) withdrew early from the trial (for reasons unrelated to the trial ulcer).

### Evidence to parametrise the model

Evidence is used to parameterise the decision model to reflect the baseline transitions between health states (i.e., transition probabilities). For each health state, evidence is identified on health-related quality of life and costs, and to inform how the intervention affects these parameters. The population characteristics are based on the TABLE trial population.

#### Baseline transition probabilities

Baseline probabilities are required relating to the simple plantar ulcer becoming complicated; the complicated ulcer requiring amputation and surgery other than amputation; healing with standard of care (i.e., saline dressings); and an ulcer recurrence on the site of a previous plantar ulcer. The model also requires the standardised mortality rate for patients with leprosy, and the operative mortality risk from amputation. We also include in the model the probability of developing a first plantar ulcer although this is not required to evaluate L-PRF, where all patients start in the simple ulcer state.

Transition probabilities between states were informed by a targeted review of the literature (see Appendix B) and data from the TABLE trial of L-PRF (see above) [22]. The review of the literature was inclusive of the full range of patients presenting with, or at risk of, neuropathic ulcers. Whilst our application looks at the specific intervention of L-PRF, we report all findings of the review as some parameters may be relevant for the assessment of other interventions for leprosy ulcers by adapting the model provided here.

#### Treatment effectiveness

To assess the effectiveness of L-PRF in improving time to ulcer healing, parametric survival modelling was used with TABLE data to predict long-term healing rates beyond the observed follow-up period in the trial. Parametric modelling uses this short-term evidence to predict long-term baseline event rates and the hazard ratio associated with the new treatment compared to baseline. Time to healing in the TABLE trial was assessed at 42 days, although patients were followed up for 70 days (or until their ulcer healed, whichever was first). After 42 days, patients were allowed to cross-over from control to treatment if their ulcer was determined to be healing slowly. This crossover was unidirectional, allowing only control group participants to switch to the L-PRF arm, while those initially receiving L-PRF remained on their assigned treatment [22]. We handled the crossover by employing an intention-to-treat approach, analysing participants based on their original randomization groups regardless of any treatment switches. While this preserves the benefits of randomization, crossover from control to intervention can attenuate the estimated treatment effect. However, only 7 participants (5.4%) switched treatment arms, and therefore any dilution of the treatment effect is unlikely to materially affect the conclusions.

Having confirmed that the assumption of proportional hazards was reasonable (see Appendix C) [23], we jointly modelled both TABLE trial treatment arms using standard survival distributions, including Exponential, Weibull, Gompertz, Log-normal, Log-logistic and Generalised Gamma [24]. The modelling framework enabled adjustment for covariables to deal with any chance imbalance between arms. It was also possible to assess how hazard ratios changed over time. The statistical fit of the model was assessed using Akaike Information Criterion (AIC) and Bayesian Information Criterion (BIC) [25], and the model was validated by clinical collaborators.

The choice of covariables is aligned with that used for the clinical effectiveness analysis [22], . As the outcome used was time to complete healing, a hazard ratio of > 1 was associated with a shorter time to healing, while a HR of < 1 was associated with a longer time to healing. The assessment of the statistical fit of the parametric survival model is reported in Appendix C.

#### Risk of recurrence

Ulcer recurrence is calculated using data on the date of patients’ six-month follow up interviews and questionnaire responses, on whether the trial ulcer had recurred on the same site. This gives the number of recurrences in trial patients and patients’ time at risk of recurrence (i.e. the time between healing and the follow up appointment) for all patients who had healed according to the blinded assessor (*n* = 99). See Appendix D for details. A relative risk of less than 1 implies that having L-PRF dressings is associated with lower likelihood of recurrence.

#### Health state quality of life

To calculate QALYs, data are needed on patients’ health-related quality of life (HRQoL, also known as utilities) in each health state. The same additional review of the literature (see Appendix B) that informed the transition parameters also searched for evidence to inform the health state utilities, but none was found. Therefore, health state utilities are estimated solely from data from the TABLE trial. HRQoL was measured using the EQ-5D-3 L instrument, which patients completed at randomisation, fortnightly until either the trial ulcer was deemed healed or trial period ended (whichever was first), and again at the six month follow up resulting in 600 EQ-5D observations. (See Appendix E for additional details.) To calculate utility scores from the EQ-5D profiles, and in the absence of a survey of public preferences from Nepal, Sri Lankan population preferences were applied [26, 27]. A generalised estimating equation (GEE) model was used to estimate the reductions in HRQoL (disutilities) associated with health states, as this model accounts for key features of the data including its distributional characteristics and repeated measures [28]. The full analysis is detailed in [11]. Since no trial participants had ulcers that became complicated and required amputation or other surgery, we were unable to estimate the HRQoL associated with a complicated ulcer or amputation directly from the trial and, instead, estimated these from the literature [29].

#### Health state costs

All costs incurred as part of the hospital stay (bed, nurse care, food), the cost of treatment with saline or L-PRF dressings, and the costs of antibiotic treatment or debridement of ulcers, if required, were included. Costs are reported in 2020 Nepalese Rupee (NRP).

The TABLE trial provided data on days in hospital (i.e., days with an unhealed ulcer) and antibiotic use, which was used to inform the costs of the simple and complicated ulcer states. To inform complete estimates of health state costs, unit costs including the daily bed charge, nursing care, food, vitamins and dressings for patients with ulcers were obtained from the trial centres. Antibiotics were given only to patients with infected ulcers and the type and course of antibiotics differed according to the relevant bacteria. Three patients required aggregate 32 days of antibiotics in the L-PRF trial arm and four patients required aggregate 30 days of antibiotics in the normal saline arm. We averaged the cost of antibiotics across all patients over time to obtain an average weekly cost.

Upon healing, patients received canvas shoes and microcellular insoles and, on average, two weeks of “self-care.” Self-care is a technique for people with leprosy to self-manage their condition and to prevent further disability. During their stay in the self-care unit, patients were trained in various aspects of self-care including the inspecting, soaking and oiling of limbs to prevent skin from drying and cracking. They were trained in the use of protective methods to use during daily activities like using cotton clothes while picking up hot utensils. This was provided as in-patient care by trained self-care staff. Patients with complicated ulcers either require amputation or another surgical procedure. These are applied in the Markov model to the transition from the complicated state to amputation or to healed, respectively. No patients in the trial required surgery or amputation, but data to inform the costs of these procedures were available from authors’ institute (see Appendix F).

### Cost-effectiveness analysis

Mean QALYs and costs per patient over the 60-year time horizon were estimated for L-PRF and its comparator. Costs were split out into those associated with hospital stay, antibiotic use and other treatments. Differential costs and QALYs were used to calculate an incremental cost-effectiveness ratio – the additional cost per QALY gained. In addition, the incremental net health benefit (iNHB) per patient and per 1,000 patients with leprosy in Nepal is reported. This is calculated as:

$$\begin{aligned} iNHB = & ({h^{L - PRF}} - {h^{saline}}) \\ &- \frac{{({c^{L - PRF}} - {c^{saline}})}}{k} \\ \end{aligned}$$

where ${h}^{L-PRF}$ is the mean per patient health gains from treatment with L-PRF, ${h}^{saline}$ is the mean per patient health benefits from treatment with saline dressings, ${c}^{L-PRF}$ is the mean per patient costs of treatment with L-PRF, ${c}^{saline}$ is the per patient costs of treatment with saline and $k$ is the marginal productivity of Nepal’s healthcare system.

iNHB represents the incremental health gained from L-PRF compared to saline, net of the health that would have been gained by devoting the same resources to other activities in the healthcare system (i.e., its opportunity cost). To calculate the opportunity cost ($k$ in the equation above), an estimate is taken from Lomas et al. (2022) of 38,970 NRP [30]. See Appendix G for details.

A positive iNHB indicates that L-PRF is cost-effective compared to normal saline. As the decision model is non-linear, it was run probabilistically. Each parameter is sampled 1,000 times from its relevant probability distribution using Monte Carlo simulation (se Table e1).

**Table 1:** Summary of the decision analytic model parameters

Parameter	Mean	SE	Probability distribution	Source	Applied in the cost-effectiveness assessment of L-PRF
**Specification of the patient population with simple ulcers**					
Baseline age (years)	53.9		Not probabilistic	TABLE trial	Yes
Ulcer area (cm^2^)	3.813		Not probabilistic	TABLE trial	Yes
**Model baseline transition probabilities (weekly)**					
Probability of developing a first plantar ulcer	0.340	0.064	Beta	Govindasamy et al., 2020 (10)	No
Probability of developing another ulcer on the site of a previous plantar ulcer (i.e., probability of an ulcer recurring)	0.022	0.003	Beta	Calculated from the TABLE trial as the number of cases where the event occurred in the non-exposed group over weeks at risk	Yes
Probability of plantar ulcer healing with standard of care (i.e., saline dressings)	0.019	0.014	Weibull	Estimated from TABLE trial	Yes
Probability of a simple ulcer becoming complicated	No evidence identified^1^				
Probability of complicated ulcer requiring amputation	0.530	0.078	Beta	Data from Anandaban Hospital patients with complicated ulcers in 2023	Yes
Probability of a complicated ulcer requiring surgery other than amputation	0.470	0.078	Beta		Yes
**Effectiveness of L-PRF**					
HR applied to healing rate for L-PRF	1.367	0.203	Weibull	TABLE trial	Yes
Relative risk of recurrence	0.740	0.352	Normal	TABLE trial	Yes
**Standardised mortality rate**					
Standardised mortality rate for leprosy	7.870		Not probabilistic	Additional mortality risk for a leprosy patient from Wang et al. (2023) applied to mortality risk by age and gender from Nepal life tables. Additional mortality risk is assumed apply equally to each age group and sex.	Yes
**Health state utilities**					
No ulcer	0.664	See Appendix E^2^		TABLE trial	Yes
Simple ulcer	0.549	See Appendix E^2^		TABLE trial	Yes
Complicated ulcer	0.498	0.52	Beta	TABLE trial, Ortegon et al., 2004 (29), see Appendix	Yes
Amputation	0.525	0.46	Beta	TABLE trial, Ortegon et al., 2004 (29), see Appendix	Yes
**Health state costs (weekly**,** 2020 NRP)**					
No ulcer/healed	0			TABLE trial assumption	Yes
Simple ulcer	8,236		Not probabilistic	TABLE trial	Yes
Healing (one off cost of shoes and insoles)	1,240		Not probabilistic	TABLE trial	Yes
Complicated ulcer	8,236		Not probabilistic	TABLE trial	Yes
Incremental cost of L-PRF	2,800		Not probabilistic	TABLE trial	Yes
**One off costs (2020 NRP)**					
Surgery other than amputation	94,128	10,300.157	Gamma	Data from Anandaban Hospital patients with complicated ulcers in 2023	Yes
Amputation	87,451	9,135.977	Gamma		Yes

^1^ This parameter is not needed for the evaluation of L-PRF as no patients in the trial had complicated ulcers

^2^ Because we used a Generalized Estimating Equations (GEE) model, the standard error is derived from the model’s covariance matrix rather than being directly calculated from individual observations. This ensures that uncertainty estimates properly account for within-subject correlations. See Appendix E

In both treatment arms, multidrug therapy (MDT) and broader leprosy disease management were assumed to be identical. The intervention effect was therefore limited to differences in ulcer healing time, recurrence risk, and associated ulcer-related costs and health-related quality of life. Incremental results should therefore be interpreted as arising from wound-care–related pathways rather than differences in underlying leprosy infection management.

#### Uncertainty analysis

Uncertainty analysis can be used to inform decision makers about the robustness of results and the likelihood of making the wrong funding decision. By running the model probabilistically, the uncertainty in each parameter is propagated through the model and jointly expressed in terms of the distribution of iNHB, which allows a calculation of the probability that L-PRF is cost-effective. Value of information methods and scenarios analyses are also used to help decision-makers understand the implications of uncertainty and identify whether investing in further evidence generation should be a priority.

##### Value of information analysis

Results from the probabilistic analysis were used to conduct Value of Information (VOI) analysis for the population. The population Expected Value of Perfect Information (EVPI) shows the total value of research that could eliminate all uncertainty associated with model parameters. As such, it gives an upper bound of the value of research and can be used to assess whether the cost of further research can be justified. We calculate EVPI per 1,000 patients.

##### Scenario and threshold analyses

Scenario analysis is undertaken to reflect uncertainty in key assumptions and the choice of evidence. This was used to assess different time to ulcer healing models (Lognormal, Loglogistic and Generalised gamma). Scenario analysis was also used to assess the implications of applying weekly dressings (as in some other studies [31, 32]) rather than twice weekly ones as in the TABLE trial. A threshold analysis was undertaken to assess how changes in the cost of hospital stay and the price of L-PRF would need to be different to alter the cost-effectiveness of L-PRF. This analysis informs the generalisability of the results to other contexts where these costs may differ.

## Results

### Model parameterisation

The literature review (See Appendix B) revealed few studies to inform the model baseline transition probabilities, state utilities or costs. Parameter estimation therefore relied on data from the TABLE trial or the authors’ institute in Nepal. Table 1 presents parameter estimates which can potentially be used in the model for the evaluation of L-PRF or other evaluations, and the sections that follow explain the sources. The rightmost column provides detail around whether the model parameter is used in the assessment of L-PRF. Those that are not may, for example, apply to different patient populations or decision problems.

#### Baseline transition probabilities

##### Probability of developing a first plantar ulcer

Govindasamy et al., 2023 provide an estimate of the probability of developing a plantar ulcer given loss of sensation in one or both feet, having not had a previous ulcer on the same site. Whilst this is not used in the assessment of the cost-effectiveness of L-PRF, since all patients in the model start with an ulcer, this value can inform models used to evaluate interventions to prevent ulcers from first occurring [10].

Probability of plantar ulcer healing with standard of care (i.e., saline dressings).

Many of the parametric models tested demonstrated a good fit to the time to healing TABLE trial data, based on an assessment of the Cox-Snell residuals, AIC, and BIC and clinical validity of the long-term extrapolations. The Weibull distribution was selected following consultation with clinicians for its simplicity, compatibility with the proportional hazards assumption, and ease of interpretation. The assessments of model fit are reported in Appendix C. In the saline arm median time to healing was 6 weeks (95% CI 5.0–7.0).

The literature review identified two studies reporting mean time to healing with standard of care. Rai et al., 2016 followed patients up for 8 weeks and reported a mean time to healing of 6.73 weeks [33]. Saraf et al. (2000) followed patients up until they healed and reported mean time to healing as 8.05 weeks [34]. More detail about the characteristics of these studies and the populations they assessed are given in Appendix B Table 1.

##### Probability of an ulcer recurring after treatment with standard care

The literature review identified only one paper, Yan et al. (2003), that reported recurrence after successful treatment with usual care, which included skin grafting, protective footwear, rest, wound dressing, and debridement [35]. Over the three-year follow up period, 1,804 patients were followed, of which 172 experienced relapses among 1,071 who healed, although time at risk (or when healing and recurrence occur) is not reported and so this paper could not be used to inform the probability of ulcer recurring parameter.

Additionally, one study that reported recurrence following surgery was identified in the review, which in principle may be useful for researchers looking to use the model to evaluate a surgical intervention. Gahalaut et al., 2005 report that 25% of patients who underwent surgery recurred within six months to one year [36]. While this study is limited in a similar way to Yan et al. (2023), it may provide useful contextual information for studies evaluating surgical interventions for leprosy ulcers.

We estimate the baseline weekly probability of recurrence from the TABLE trial of 0.022 (0.003). This value may differ in other contexts if, for example, at risk individuals did more (or less) subsistence agriculture and/or were on their feet more (or less), had better (or worse) access to protective footwear, or checked the bottoms of their feet more (or less) frequently for wounds.

##### Probability of a simple ulcer becoming complicated

No literature was found to inform the probability of a simple ulcer becoming complicated, and no patients in the TABLE trial went on to have complicated ulcers, and so this probability is assumed to be zero.

##### Probability of complicated ulcer requiring amputation or other surgery

The literature review did not identify any studies that could inform the probability of a complicated ulcer requiring amputation or the probability of a complicated ulcer requiring surgery other than amputation. These were instead informed from data from authors’ institute as the proportion of complicated ulcers which required amputation (single or multiple digit, forefoot or below knee) versus other surgery (calcaneus paring, osteotomy or sequestrectomy) in 2023.

#### Treatment effectiveness

##### Probability of plantar ulcer healing with L-PRF treatment

This was estimated alongside the probability of plantar ulcer healing with standard of care (i.e., saline dressings) from the TABLE trial (see above). In the L-PRF arm of the TABLE trial median time to healing was 5.6 weeks (95% CI 4.4–6.6). The hazard ratio of healing is applied to the transition rate for L-PRF to derive the rate of healing for standard of care, which is then translated into a transition probability.

##### Probability of an ulcer recurring after treatment with standard care

We estimate a relative risk of recurrence of 0.74 (95% CIs 0.04, 1.43) implying a 26% reduction in the rate of recurrence for patients treated with L-PRF compared to those treated with saline. See Appendix D for more details.

##### Mortality

Nepali life tables provided data on death rate by age and sex. Using data from Wang et al. (2023) on the additional mortality burden among leprosy patients in Chongqing, China, we adjust the Nepali mortality rates for each age and sex using the yearly relative standardised mortality rate, and then convert these into weekly probabilities. Mortality was not included in the literature review to identify parameters, but Wang et al. (2023) was identified separately and included [37].

Input from clinicians suggests that the additional risk of mortality associated with amputation and other surgery in patients with leprosy is negligible, as significant additional mortality risk arises from vascular complications that can lead to poor wound healing, which is not present in leprosy. Consistent with this, Sousa Macedo et al. (2021) found that patients with insensitivity due to diabetes have higher rates of second or subsequent amputations performed on the same limb after an initial amputation and other complications compared to other subgroups (for example, leprosy) [38]. Therefore, no operative mortality risk from amputation due to leprosy is applied in the model.

### Health-related quality of life

Health state utilities are reported in Table 1. The literature review identified studies reporting HRQoL for patients with leprosy, but no values specific to leprosy ulcers [12, 39, 40]. Therefore, model health state utilities for the ulcer and no ulcer health states were solely taken from the TABLE trial (Table 1). The estimated mean utility using our regression model for the sample across all time points was 0.528 for patients with an ulcer and 0.664 with a healed ulcer, controlling for age and sex. Health state utilities for complicated ulcers and amputations draw upon data from [29]. See Appendix E for detailed calculations.

### Resource use and costs

Weekly costs are the same for all patients in the trial apart from the acquisition cost of L-PRF compared to normal saline, and antibiotic use. We find a weekly cost of 8,236 (2020 NPR) for treating patients in the simple ulcer state or the complicated ulcer state with saline dressings. There is an additional cost of 2,800 (2020 NRP) that applies to both states when patients are treated with L-PRF. A one-off cost of 1,240 (2020 NRP) is incurred for shoes and microcellular insoles when patients have healed. After this, we assume there to be no cost when there is no ulcer present. Surgery other than amputation and amputation incur one-off costs of 94,128 and 87,451 (2020 NRP), respectively. As the weekly costs were derived from hospital data that reported a consistent unit cost across patients without any information on between-patient variability, we were not able to assign an uncertainty distribution to the weekly cost for each state in the probabilistic sensitivity analysis. While length of stay varied between individuals affecting overall cost per patient, this variation is captured in the model’s transition probabilities.

### Cost-effectiveness of L-PRF

Table 2 reports incremental mean costs, effects and incremental mean NHB (per person and per 1,000 patients). This is based on the estimate of opportunity cost of healthcare expenditure in Nepal that every 38,970 NRPs spent on healthcare in Nepal generates one QALY in the wider healthcare system. L-PRF improves HRQoL, but incurs an additional cost. The cost of treatment itself (L-PRF dressings) is higher than the cost of saline dressings, but patients who are treated with L-PRF incur lower hospital costs because they heal more quickly with a resulting incremental cost of L-PRF of 6,731 (95% CI -58,627–63,563). The difference in mean QALYs between the groups is small at 0.006 per patient (95% CI − 0.004–0.019). The resulting incremental cost effectiveness ratio is 1 million NRP per QALY gained. This means that L-PRF generates health at a worse rate, on average, (1 million NRP per QALY gained) than the Nepali healthcare system generates health (38,970 per QALY gained), and is not cost-effective (albeit there is uncertainty around both the mean incremental costs and incremental benefits, see below).

**Table 2:** Total mean costs and QALYs of treatment per patient (costs in 2020 Nepalese Rupees)

	Standard	Hospitalisation	Antibiotics	Saline treatment	L-PRF	Hospitalisation	Antibiotics	L-PRF treatment	Mean difference
**Mean cost**	**125,092**	104,520	34	11,248	**131,824**	83,818	27	40,590	6,731
**95% CIs**	**102**,**040**	84,962	28	9,143	**69**,**038**	45,483	15	22,026	-58,627
	**151**,**924**	127,897	42	13,763	**194**,**448**	122,197	40	59,175	63,563
**Mean QALYs**	4.013				4.019				0.006
**95% CIs**	3.766				3.772				-0.004
	4.239				4.253				0.019
Incremental cost per QALY gained							1,093,373		
Probability of being cost effective at a threshold of 38,970 (2020 Nepalese rupees)							0.41		
**Incremental net health benefit at a threshold of 38**,**970 (2020 Nepalese rupees)**									
Incremental net health benefit per patient from L-PRF							− 0.167 (-1.64, 1.52)		
Incremental net health benefit from L-PRF per 1,000 patients							-167 (-163,643, 151,929)		

* Totals reflect the mean of total costs (or QALYs) calculated within each Monte Carlo simulation; therefore, the sum of mean component values may not equal the reported mean total

**95% confidence intervals were calculated from the Monte Carlo simulation and reflect the non-parametric distribution of cost and QALYs

*** Values per 1,000 patients are obtained by scaling the per-patient Monte Carlo outputs by 1,000 (means and percentile intervals scaled accordingly)

Although the average patient receiving L-PRF would have better a better health outcome (by 0.006 QALYs on average compared to if they received saline), the additional cost associated with providing L-PRF to that patient (6,731 NRP on average) could have generated 0.173 QALYs if spent in the wider healthcare system. Based on the estimated marginal productivity of 38,970 NRP per QALY, this additional expenditure corresponds to approximately 0.173 QALYs forgone in the wider system. When these opportunity costs are accounted for, the incremental net health benefit is − 0.167 QALYs per patient, or approximately 167 QALYs per every 1,000 patients.

#### Uncertainty analysis

These mean costs and benefits are estimated with uncertainty. Based on the probabilistic analysis, L-PRF is unlikely to be cost-effective at a threshold of 38,970 NRP (with a probability of being cost-effective of around 0.41). Appendix G reports results scenarios applying the lognormal, loglogistic and generalised gamma models to estimate time to ulcer healing.

**Fig. 2:**
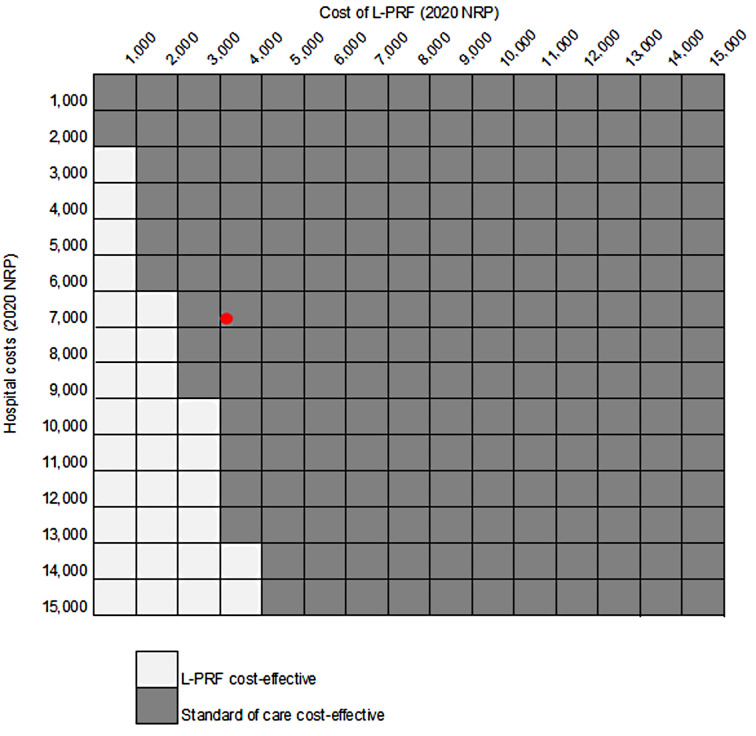
Threshold analysis of hospital and L-PRF costs

A threshold analysis was undertaken to assess how much hospital stay costs and the acquisition price of L-PRF would need to change to alter whether L-PRF was deemed cost-effective or not. L-PRF currently has a price of 3,600 NRP (2020) per week in Nepal (provided twice weekly) and the cost of a week stay in hospital is 7,434 NRP (2020) per week (as indicated by the red dot). If it was feasible that L-PRF was equally as effective if applied only once weekly, reducing its cost by half, it would still not be cost-effective. As shown in Fig. 2, L-PRF is cost-effective when hospital costs are higher as its slightly faster healing rate means that less time is spent in hospital. This is, however, offset if L-PRF costs are higher. (The red dot represents the cost of a week in hospital and weekly L-PRF acquisition costs from the TABLE trial.)

##### Value of information analysis

The population EVPI is $133,130 per 1,000 patients. Conservatively assuming the research would benefit five years of future patients in Nepal, the population EVPI is $627,987. Assuming this research could benefit five years of patients around the world living with leprosy and at risk of ulcers, the global EVPI is $87.5 million. This represents the upper bound of the value of this research to five years of future patients, as it assumes that the research would resolve all uncertainty.

## Discussion

To our knowledge, this is the first decision analytic model for the economic evaluation of interventions to manage neuropathic ulcers arising from leprosy in LMIC.

The Markov model reflects relevant differences in patients’ health status, progression, or outcomes, and was developed with clinicians to ensure that the states are clinically meaningful. It can be used to assess interventions for preventing or treating plantar ulcers in patients with loss of sensation in their feet resulting from leprosy, or the major sequelae from such ulcers: infections, amputations and other surgery. The use of the model to inform decisions on the value for money of interventions to treat and prevent leprosy ulcers is demonstrated through its application to L-PRF, a novel wound healing intervention provided in a hospital setting. Applying the model to assess different interventions, particularly those undertaken outside of a hospital setting, requires careful assessment of the transferability of the parameters and potentially the re-estimation of some parameters to ensure they are contextually relevant.

We have endeavoured to compile all available evidence to inform transition probabilities for a range of decision questions in neuropathic ulcers arising from leprosy. Nonetheless, gaps in the evidence-base remain, and trial data may continue to form a critical component to parameterising the model for other decision questions. One advantage of using data from a randomised controlled trial is that using a single source ensures consistency. The model has largely been parameterised from trial data, and reflects a specific hospital setting. A limitation of this is that its generalisability is limited to other hospital settings. As with the probability of a simple ulcer becoming complicated, some other probabilities may also differ in the community setting. For example, a simple ulcer may heal without treatment (e.g., if individuals offload pressure and/or undertake basic wound care). To some extent this was mitigated through the sensitivity analysis varying the cost of the intervention and the hospital inpatient costs, although this relates to the effectiveness of this specific intervention.

We demonstrated how the model can be used by the application to L-PRF, where patients start in the simple ulcer state. The transition probabilities used in the model are largely drawn from the TABLE trial of L-PRF versus standard of care, reflecting a dearth of good quality studies in LMIC. Our model assumes that the probability of death among patients with leprosy is higher than in the age-gender adjusted general population based on data from Wang et al. (2023); however, it is plausible that the risk of death differs between model states.

Our model uses health state utilities estimated using data from the TABLE trial for the simple ulcer and no ulcer states. While the preference data necessary to transform patient’s HRQoL responses to the EQ-5D-3 L are not available for Nepal, they have been estimated in Sri Lanka where mean EQ-5D-3 L utility value is 0.867 [26]. Mobility is noted to be important to the utility score in Sri Lanka, which the authors link to the lower health and social support for people with mobility impairments in Sri Lanka [26]. This finding is consistent with other studies in the region [41, 42], and therefore is also likely to be relevant in Nepal. We estimate a mean EQ-5D-3 L utility value of 0.664 for leprosy patients with no ulcers. This is consistent with the literature, which finds that leprosy patients have lower HRQoL than the general population [43–46]. For the complicated, amputation and amputee health states, we apply data from a paper assessing DFU in a high-income country to the baseline HRQoL to that of the patients in our trial, and this should be updated when better data is available. A more detailed analysis of utilities based on the TABLE trial is available in Gibbs et al. (2025), with potential scope to adjust the HRQoL to other populations [11].

To estimate time-to-healing with twice weekly saline dressing changes and twice weekly L-PRF dressing changes, we implemented parametric survival modelling. This enabled us to extrapolate time to healing beyond the trial follow-up and calculate the relative risk of recurrence. The estimated hazard ratio for healing at 42 days from the clinical trial was 1.3 (SE 0.8–2.0) using Cox Proportional Hazards [22]. We replicated this at 42 days, but made use of the full 70 days of follow up data for our analysis. To utilize the full 70-day follow-up data and enhance the precision of our healing rate estimates, we included all available data in our analysis. Notably, even if we had restricted our analysis to the 42-day data, L-PRF would still not be considered cost-effective, indicating that our conclusions remain robust regardless of the follow-up period analysed.

There is uncertainty in a range of parameters, including the effect of L-PRF on time to healing. Consistent with the results of the clinical trial [22], the Weibull model estimates showed wide confidence intervals, indicating substantial uncertainty about the true direction of effect. Resolving this uncertainty could significantly improve decision-making in clinical practice and policy. To quantify the potential benefit of further research, we calculated the EVPI, a measure of the maximum value that could be gained if all uncertainty were resolved. For a cohort of 1,000 patients in a given year in Nepal, the EVPI was $133,130. However, 86% of leprosy cases are in India, Brazil, Indonesia, Nigeria, Ethiopia, Bangladesh, Democratic Republic of the Congo, Nepal, the Philippines, and China [47]. As such, further studies to improve clinical and policy decisions regarding leprosy ulcer management could potentially have substantial benefits to patients across the world and in future years.

In the current model, weekly costs for simple and complicated ulcers were assumed to be similar, reflecting hospital accounting data indicating comparable bed, nursing, and dressing costs in the hospital setting informing this analysis. Additional costs associated with complicated ulcers were applied at the point of surgery or amputation. This approach may understate the economic burden of prolonged or medically managed complications if these incur sustained additional resource use beyond that observed in the trial setting. If complicated ulcers generate higher ongoing costs in other contexts, the potential cost offsets associated with faster healing may be greater than estimated in our base case. These considerations should be borne in mind when interpreting the cost-effectiveness results and adapting the model to other settings.

We did not assign an arbitrary uncertainty distribution to the cost parameters in the model, and instead undertook a threshold analysis to assess how variation in key costs, specifically, the price of L-PRF and hospital stay costs, would affect cost-effectiveness. This approach avoids speculative assumptions and instead identifies the cost values at which the intervention would be considered cost-effective. The analysis supports the generalisability of the results to settings where these costs may differ from those observed in the trial. However, as a result of not assigning uncertainty distributions to the cost parameters, the EVPI may be underestimated if, in reality, there is substantial uncertainty in these cost parameters.

The analysis of L-PRF found that the health that could have been gained by spending the money required to provide L-PRF on existing funded healthcare in the Nepali healthcare system outweighed the incremental health gains achieved by providing L-PRF (compared to saline treatment). The estimate of the health that could have been generated with the additional money required to provide L-PRF (net of saline), is based on an estimate of the marginal productivity of the Nepali healthcare system, and represents opportunity cost in that system. Using this estimate implies that money spent by non-government groups aiming to improve health should generate health at a rate at least as good as that of government funding for health. However, these results rely on the parameterisation and opportunity cost estimate applied in this setting. Uncertainty remains around key inputs, including transition probabilities and costs, and alternative estimates of health opportunity cost or local epidemiology could alter the conclusion.

To avoid double-counting in our model, which extrapolates beyond the duration of the trial, we do not include data collected by the trial team regarding follow-up visits. Our analysis accounts for the costs associated with these through the inclusion of the ulcer recurrence parameter, which differs between treatment arm. We favoured this over using the data collected by the trial team since their data is not explicitly linked to the trial ulcer, which makes it incompatible with the conceptual basis for our decision model. Nonetheless, the trial data suggest a lower number of follow-up visits among patients treated with L-PRF, although this difference was not statistically significant, consistent with the non-significant reduction in recurrence rates observed in the L-PRF arm.

While the model can be used to evaluate interventions where patients start in any of the health states, apart from amputee and death (the absorbing states), application beyond the current context would require re-parameterisation and, in some cases, structural modification. For example, prevention-focused strategies would require estimation of first-ulcer incidence (reported in Table 1, but not used in the analysis of L-PRF); community-based interventions may require alternative healing probabilities and cost estimates; and earlier-stage neuropathy populations may warrant distinguishing between ulcer-naïve (at risk of first ulcer) and previously healed individuals (at risk of recurrence), as these groups have different baseline risks and transition probabilities. In particular, the definition of the complicated ulcer state reflects surgical management pathways observed in the hospital setting, where deep tissue or bone involvement (including osteomyelitis) typically proceeds to operative intervention. The absence of progression to complicated ulcers in the TABLE trial is clinically reassuring and likely reflects the hospital-level care context. While this limits direct empirical estimation of the transition probability within the trial dataset, the inclusion of the complicated ulcer state ensures that the model remains structurally appropriate for settings where severe progression may be more common. In settings where medically managed severe infection represents a distinct and resource-intensive pathway with different long-term consequences, further refinement of this state structure may be appropriate. The model could also be extended to allow for greater flexibility; for example, evaluation of a trial where amputations do occur may want to apply tunnel states in the model reflecting different costs and outcomes (e.g., risk of death) in the weeks following an amputation. The Markov trace is equipped to incorporate this. Additionally, the model can be used to perform a VOI analysis, making it a potentially valuable tool for planning trials and guiding related data collection efforts.

## Conclusion

This paper provides a decision analytical state transition model of a discrete time Markov structure for the economic evaluation of interventions to manage neuropathic ulcers arising from leprosy. Applied to L-PRF in a hospital setting in Nepal, the model suggests that the intervention is unlikely to be cost-effective under the parameterisation and health opportunity cost estimate used. However, important uncertainty remains and the evidence base to inform key parameters is limited. Although we have complied data through reviews of the available literature, trial data, and clinical input to inform the model, data gaps persist. As such, researchers intending to use the model to evaluate an intervention should carefully consider whether the parameters provided here are relevant to their context and update these where possible and necessary.

## Supplementary Information

Below is the link to the electronic supplementary material.

Supplementary Material 1

## Data Availability

The model file is available on request from the authors.

## Abbreviations

AIC: Akaike Information Criterion
BIC: Bayesian Information Criterion
DFU: Diabetic Foot Ulcer
EQ-5D-3 L: EuroQol 5-Dimensions, 3-Level instrument
EVPI: Expected Value of Perfect Information
GEE: Generalised Estimating Equations
HR: Hazard Ratio
HRQoL: Health-Related Quality of Life
iNHB: Incremental Net Health Benefit
LMIC: Low– and Middle-Income Country
L–PRF: Leukocyte– and Platelet-Rich Fibrin
MDT: Multidrug Therapy
NHB: Net Health Benefit
NHRC: Nepal Health Research Council
NIHR: National Institute for Health and Care Research
NRP: Nepalese Rupees
NLEP: National Leprosy Eradication Program
QALY: Quality-Adjusted Life Year
SE: Standard Error
MRI: Magnetic resonance imaging
TABLE trial: Trial of Autologous Blood products, leukocyte and platelet-rich fibrin (L-PRF), to promote ulcer healing in LEprosy
VOI: Value of Information
WHO: World Health Organization
