# Supplementary material for: Modelling the cost-effectiveness of interventions to treat or prevent neuropathic ulcers arising from leprosy: application to L-PRF
Source: Cost Eff Resour Alloc. 2026 Mar 26;24:61. doi: 10.1186/s12962-026-00739-8 (PMC13141576; doi:10.1186/s12962-026-00739-8)
Supplement: Supplementary file 1 — Supplementary Material 1 [file 12962_2026_739_MOESM1_ESM.docx]

# Appendices

[Appendices 1](#_Toc221894206)

[Appendix A. Systematic Review of Model‑Based Economic Evaluations of Prevention and Treatment Interventions for Leprosy Ulcers 4](#_Toc221894207)

[Methods 4](#_Toc221894208)

[Appendix A Table 1. Inclusion and exclusion criteria 4](#_Toc221894209)

[Results 5](#_Toc221894210)

[Appendix A Figure 1. PRISMA Flow Diagram 6](#_Toc221894211)

[Appendix A Table 2. Study characteristics 7](#_Toc221894212)

[Key findings 9](#_Toc221894213)

[Limitations 9](#_Toc221894214)

[Search strategy 11](#_Toc221894215)

[Philips checklist methodological quality assessment 16](#_Toc221894216)

[CHEERs checklist quality assessment 19](#_Toc221894217)

[Appendix B. Literature searches to inform decision model parameters 21](#_Toc221894218)

[Search 1: Clinical parameters 21](#_Toc221894219)

[Appendix B Figure 1: Prisma of literature search for clinical parameters to inform the model 22](#_Toc221894220)

[Appendix B Table 1: Ulcer healing 23](#_Toc221894221)

[Appendix B Table 2: Ulcer recurrence 24](#_Toc221894222)

[Search 2: Health-related quality of life 24](#_Toc221894223)

[Appendix B Table 3: iHME 2019 DALY weights for disfigurement due to leprosy 25](#_Toc221894224)

[Search 3: Cost and resources use 26](#_Toc221894225)

[Appendix B. Search 1: Clinical parameters search strategy 27](#_Toc221894226)

[Appendix C. Time to healing 30](#_Toc221894227)

[Data 30](#_Toc221894228)

[Assessment of model fit 30](#_Toc221894229)

[Appendix C Figure 1. Kaplan-Meier Survival Curve 30](#_Toc221894230)

[Appendix C Figure 2. Cox-Snell residuals 31](#_Toc221894231)

[Appendix C Table 1. Estimated coefficients, Akaike information criterion, and Bayesian information criterion 31](#_Toc221894232)

[Appendix C Figure 3. Modelled survival truncated at 52 weeks 32](#_Toc221894233)

[Weibull model 33](#_Toc221894234)

[Appendix C Table 2. Weibull Proportional Hazards Model for Time to Healing in Patients with Plantar Ulcers 33](#_Toc221894235)

[Appendix C Table 2. Cholesky Decomposition Matrix for Covariance Structure in the Weibull Proportional Hazards Model 34](#_Toc221894236)

[Log-logistic model 35](#_Toc221894237)

[Appendix C Table 3. Log-logistic Accelerated Failure Time Model for Time to Healing in Patients with Plantar Ulcers 35](#_Toc221894238)

[Appendix C Table 4. Cholesky Decomposition Matrix for Covariance Structure in the Log-logistic Accelerated Time Failure Model 36](#_Toc221894239)

[Lognormal model 37](#_Toc221894240)

[Appendix C Table 5. Lognormal Accelerated Failure Time Model for Time to Healing in Patients with Plantar Ulcers 37](#_Toc221894241)

[Appendix C Table 6. Cholesky Decomposition Matrix for Covariance Structure in the Lognormal Accelerated Time Failure Model 38](#_Toc221894242)

[Generalised gamma model 39](#_Toc221894243)

[Appendix C Table 7. Generalised Gamma Accelerated Failure Time Model for Time to Healing in Patients with Plantar Ulcers 39](#_Toc221894244)

[Appendix C Table 8. Cholesky Decomposition Matrix for Covariance Structure in the Generalised Gamma Accelerated Time Failure Model 40](#_Toc221894245)

[Appendix D. Risk of recurrence 42](#_Toc221894246)

[Appendix E. Health state utilities 43](#_Toc221894247)

[Literature review 43](#_Toc221894248)

[Ulcer and no ulcer 43](#_Toc221894249)

[Appendix E Table 1: Baseline clinical and demographic characteristics: n, (%) 44](#_Toc221894250)

[Appendix E Table 2. Mean EQ-5D (se) recorded at each time point with numbers of measurements taken 44](#_Toc221894251)

[Appendix E Table 3: GEE GLM results with disutility as dependent variable, obs = 593 45](#_Toc221894252)

[Complicated ulcer and amputation 46](#_Toc221894253)

[Appendix E Table 4: Calculation of health state utilities using decrements from Ortegon et al (2004) 46](#_Toc221894254)

[Appendix F. Costs and resource use 47](#_Toc221894255)

[Appendix F Table 1. Costs and resource use associated with ulcer healing 47](#_Toc221894256)

[Appendix F Table 2. Costs and resource use for patients with ulcers 47](#_Toc221894257)

[Appendix F Table 3. Antibiotic costs 48](#_Toc221894258)

[Appendix F Table 4. Amputation and other surgery costs (2023 NRP) 49](#_Toc221894259)

[Appendix G. Opportunity cost 50](#_Toc221894260)

[Appendix H. Results of L-PRF using the other three models 50](#_Toc221894261)

[Appendix H Table 1. Incremental net health benefit by model (probabilistic sensitivity analysis) 50](#_Toc221894262)

[Appendix H Table 2. Total costs and effects of treatment (Weibull probabilistic sensitivity analysis) 50](#_Toc221894263)

[Appendix H Table 3. Total costs and effects of treatment (Lognormal probabilistic sensitivity analysis) 51](#_Toc221894264)

[Appendix H Table 4. Total costs and effects of treatment (Loglogistic probabilistic sensitivity analysis) 51](#_Toc221894265)

[Appendix H Table 5. Total costs and effects of treatment (Generalised gamma probabilistic sensitivity analysis) 52](#_Toc221894266)

[References 52](#_Toc221894267)

###

### Appendix A. Systematic Review of Model‑Based Economic Evaluations of Prevention and Treatment Interventions for Leprosy Ulcers

A review of all published model-based economic evaluations pertaining to the treatment and prevention of leprosy ulcers, diabetic foot ulcers (DFU) and Buruli ulcers in a LMIC setting was conducted. The purpose of this review was to contribute to a greater understanding of how these model-based economic evaluations are conducted in order to help inform future decision models for interventions targeted at leprosy ulcers.

#### Methods

The search strategy for this review was defined by developed by the Centre for Reviews and

Dissemination at the University of York. A literature search was performed, spanning from 2000 to 18th May 2020. The retrieved studies were then screened against the inclusion and exclusion criteria (see Appendix A Table 1). A decision model was defined as a set of mathematical relationships between health states or pathways, characterising the range of possible disease prognoses, and the impacts of alternative interventions (1). Hence, studies were excluded if they did not use a decision analytic model, such as if the cost-effectiveness analysis was nested or performed on the back of clinical trial findings.

##### Appendix A Table 1. Inclusion and exclusion criteria

| **Inclusion criteria** | |
| --- | --- |
| Population | Individuals with or at risk of DFU, Buruli ulcers or leprosy ulcers |
| Interventions | Treatment or prevention |
| Study design | Cost-effectiveness analysis, cost-utility analysis or cost-consequence analysis, which used a standalone decision analytic model |
| Language | English or Portuguese |
| Timeframe | From 2000 to 18 May 2020 |
| **Exclusion criteria** | |
| Population | Individuals with a pressure ulcer or an ulcer not attributed to diabetes, Buruli or leprosy |
| Interventions | Diagnostic tests |
| Study design | Cost-benefit analysis, cost-minimisation analysis, or any study that did not include a decision analytic model |

The search was conducted in MEDLINE ALL (Ovid), Embase (Ovid), EconLit (Ovid), CINAHL Complete (Ebsco), NHS Economic Evaluations Database (NHS EED), HTA Database and the Cost Effectiveness Analysis (CEA) Registry.

EndNote software was then used to manage the search results and remove duplicates. After duplicates had been removed, a total of 2832 papers were eligible for further screening. The search strategy itself was developed by an information specialist using Ovid MEDLINE. Various terms for diabetic, leprosy and Buruli ulcers were combined with a search strategy for identifying economic evaluations, developed by the Centre for Reviews and Dissemination. The search strategy identified studies from both subject headings and within text.

Study selection occurred in three stages and was performed by the main reviewer (HS) under supervision by the two other reviewers (RF and JO). The three stages consisted of a preliminary title screening followed by an abstract screening prior to a final a full text screening. At the title and abstract screening stage, studies were only excluded if they specifically met any of the exclusion criteria or it was obvious that they did not satisfy the inclusion criteria. The full texts of the remaining studies were then screened against the inclusion/exclusion criteria (apart from the country criterion) to assess their eligibility for inclusion in the review. The inclusion of articles which the main reviewer was uncertain about were discussed and decided among the team. Lastly, the country criterion was applied.

The data extraction sheet was constructed by the main reviewer (HS) and then refined through discussion between all three reviewers (RF, JO, HS). The data extracted concerned the study characteristics; patient characteristics, such as details of the wound/ulcer; features of the decision-analytic model; clinical inputs and outcomes; cost inputs and outcomes; study results and the authors conclusions of the study. Any disagreements were resolved by discussion.

The methodological quality of each paper was assessed using the Philips checklist (2) (see below section “Philips checklist methodological quality assessment”). Quality assessment for reporting was carried out using the Consolidated Health Economic Evaluation Reporting Standards (CHEERS) checklist. The CHEERS checklist was designed specifically to optimise the reporting of health economic evaluations (Husereau et al 2013). Each study was assessed against the 24-point checklist, receiving a score of 0, 0.5 or 1 pertaining to whether the study didn’t did not fulfil, partially fulfilled or fully fulfilled each of the criteria. These scores were then aggregated to produce an overall quality assessment score given as a percentage of the complete checklist. Any checkpoints that were not applicable to an individual study were removed from the final score calculation. The quality assessment was completed solely by the main reviewer (HS). The quality assessment is detailed in the below section “CHEERs checklist quality assessment”. For transparency, the page/pages relating to where the study fulfilled each of the criteria was also reported.

#### Results

The literature search returned a total of 4777 studies. After removing duplicate papers and initial screening of titles and abstracts, 491 full texts were retrieved and assessed for eligibility. Subsequent to full-text screening, 3 studies were included within the review. This study selection process is summarised in the PRISMA diagram in Appendix A Figure 1. Of note, 47 studies met all the inclusion/exclusion criteria with the exception of a being set in a LMIC.

##### Appendix A Figure 1. PRISMA Flow Diagram


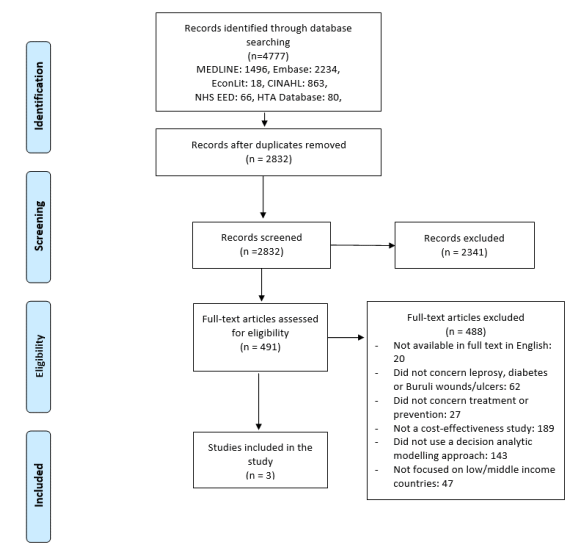


From among the studies included, a total of 3 models were identified. These studies were conducted by Wu et al (3), Romero Prada et al (4) and Cárdenas et al (5), set in China, Colombia and Peru respectively, all of which are upper-middle income countries. All three studies pertained to DFU. Two of the studies used a Markov structure whilst the remaining study took a decision tree modelling approach. The interventions considered included both prevention and treatment interventions, each of which were found to be cost-effective or cost-saving by the author of the study. The methodological quality of the papers was low, particularly with regards to the model structure and data identification and incorporation process.

Incremental costs and clinical outcomes were reported for each intervention and comparator and were subsequently used to calculate ICERs as a measure of cost-effectiveness within all three studies. In all three studies, the comparator of ‘standard’ or ‘sub-optimal’ care was used as the anchor point for ICER calculations. As only Wu et al (3) and Romero Prada et al (4) reported a cost-effectiveness threshold, the overall cost-effectiveness of the intervention/s could only be assessed in these two studies. These cost effectiveness thresholds varied significantly with Wu et al (3) utilising a threshold of US$8382 equal to Chinas GDP in 2016, whilst Prada et al [17] a compares ICERs to a threshold of US$53,088, three times Colombia’s GDP in the same year.

Wu et al (3) and Prada et al (4) both found the evaluated interventions to be cost-effective when compared to standard care and with reference to the cost-effectiveness threshold. Wu et al (3) generated a negative ICER indicating their intervention approach was both more effective and cost saving compared to standard care. As Cárdenas et al (5) did not formally state a cost-effectiveness threshold, they were unable to draw concrete conclusions as to the cost-effectiveness of their interventions. That said, their intervention of ‘standard care’ was cost-saving when compared to ‘sub-optimal’ care.

All three studies conducted sensitivity analysis to account for uncertainty within the parameters of the model. Wu et al (3) and Romero Prada et al (4) undertook probabilistic sensitivity analysis using Monte Carlo simulation whilst a Deterministic sensitivity analysis was carried by Cárdenas et al (5). Both Wu et al (3) and Cárdenas et al (5) used a univariate approach, whilst Romero Prada et al (4) conducted a multivariate analysis. Cárdenas et al (5) found the most sensitive variable to be the cost of the interventions whilst Wu et al (3) found the risk ratio (RR) of foot ulcers and amputation to have the biggest impact on the cost-effectiveness of the intervention.

Each of the studies reported limitations. All three reported as one limitation, a lack of available data on clinical outcomes due to an inadequate number of clinical trials conducted in each of the middle-income countries in which the economic evaluation was set. A second shared limitation was the need for simplifying assumptions to be made. Specifically, Wu et al (3) assumed that only one ulcer could occur at any one time, whilst Cárdenas et al (5) stated that they had assumed patient’s adherence to treatment was 100%. Romero Prada et al (4) also acknowledged that they had not considered the possibility of hospital re-admission. Both Wu et al (3) and Romero Prada et al (4) also stated their studies were limited by the interventions they had chosen, restricting the study’s application to real world scenarios however both studies again put this down to a lack of country specific data on other interventions that would have been appropriate for use in their study.

##### Appendix A Table 2. Study characteristics

|  | Wu et al (2018) | Romero Prada et al (2018) | Cardenas et al (2015) |
| --- | --- | --- | --- |
| **Study population** | | | |
| **Study type** | Cost-utility | Cost-utility | Cost-effectiveness |
| **Country** | China | Colombia | Peru |
| **Perspective** | Healthcare payer | Healthcare payer | Societal |
| **Wound restrictions** | Diabetic | Diabetic, Wagner’s 3 & 4 diabetic ulcers, controlled infection | Diabetic, no ischemia |
| **Population restrictions** | Type 2 diabetic, newly diagnosed | none | Type 2 diabetic, high-risk |
| **Interventions and comparators** | | | |
| **Clinical setting** | Hospital | Hospital | Hospital and home |
| **Intervention type** | Prevention and Treatment | Treatment | Prevention |
| **Specific interventions** | Guideline based-optimal care programme (diabetic sock, education, topical hydrogen, pressure off loading footwear, Pharmacological therapy) | Human epidermal growth factor (rhEGF) | Standard care: (bi-monthly consultations, a higher frequency of tests, podiatrist consultation with foot evaluation, education and use of insoles), Optimal care: standard care + self-monitoring of foot temperature |
| **Comparator** | Usual care | Conventional treatment | sub-optimal care: an annual visit to a physician and podiatrist where routine tests are performed |
| **Decision analytic model** | | | |
| Model type | Markov | Markov | Decision Tree |
| Time horizon | 5 years | 5 years | 1 year |
| Cycle length | 1 month | 1 week | n/a |
| Number of health states/outcomes | 8 | 5 | 14 |
| **Clinical data** | | | |
| Primary outcome measure | QALYs | QALYs | Number of deaths averted |
| Other outcome measures | Number of minor amputations averted, number of major amputations averted, Expected Life Years (Lys) | Number of amputations averted | Number of amputations averted |
| Data source | Literature | Literature | Literature |
| Discount rate | 5% | 5% | n/a |
| **Cost data** | | | |
| Direct input costs | Intervention, comparator, inpatient, outpatient | Intervention, comparator, inpatient | Intervention, comparator, inpatient, outpatient |
| Indirect input costs | n/a | n/a | Human capital costs (due to premature death) |
| Data source | Local and literature | Local and Literature | Local and Literature |
| Currency | USD and CNY | USD and COP | USD and PEN |
| Discount rate | 5% | 5% | n/a |
| **Results** | | | |
| Measure of Cost-effectiveness | ICERs | ICERs | ICERs |
| Cost-effectiveness of Intervention | Cost-effective and cost-saving | Cost-effective | Cost-saving |
| Cost-effectiveness Threshold | $8382 | $53,088 | n/a |
| Sensitivity analysis | Univariate, Probabilistic | Multivariate, Probabilistic | Multivariate, Deterministic |
| Scenario analysis | none | none | none |

##### Key findings

This systematic review identified a deficit of any full cost-effectiveness or cost-utility studies that compare interventions to treat or prevent leprosy ulcers, both in LMICs and in HICs, between the years 2000 and 2020. This finding is consistent with the findings of Veen et al (6), who reviewed cost-effectiveness studies on the prevention of leprosy related disabilities in 2016. Veen et al’s review identified three cost-effectiveness studies, of which two were within-trial cost-effectiveness studies (7,8). The other study identified was model-based and focused on the cost-effectiveness of leprosy case detection, treatment and prevention of disability. However, the latter study failed to compare alternative interventions for the treatment or prevention of ulcers (9). On this basis, the three studies identified by Veen et al et al were excluded from this review. Veen et al’s study was published over eleven years prior to this review and since, there have been no further reviews published that consider economic evaluations for the prevention or treatment of leprosy ulcers.

To evaluate a greater number of healthcare technologies that are appropriate for use on leprosy wounds, this review also considered economic evaluations concerning DFUs and Buruli ulcers in LMICs. This strategy retrieved just three studies, all of which evaluated medical interventions for use on DFUs in upper-middle income countries. As a result, another key finding of this review is the lack of full economic evaluations that pertain to the prevention and treatment of neuropathic ulcers more generally in a LMIC settings.

The three studies that were retrieved for inclusion in this review were based in China, Colombia and Peru, respectively.

With regards to the model structure, only Wu et al (3) provided a model that sufficiently captured the natural progression of DFUs. Romero Prada et al (4) did not model for infected ulcers despite their relevance to the intervention being assessed. Cárdenas et al (5) used a decision tree model which was less suitable for modelling the progression of DFUs.

Each study faced limitations with regard to data. Although a clear lack of relevant sources was apparent within each study, this was exacerbated by a lack of transparency in the data identification and incorporation process.

##### Limitations

We excluded studies that were not published in English or Portuguese due to the resource limitations of this review. South American studies made up the majority of inaccessible but relevant literature for this review. In particular, an economic evaluation published by Collazo Herrera et al (10), into the use of the cost-effectiveness of Heberprot P treatment for diabetic foot ulcers in Havana fulfilled all of the inclusion criteria in this review and would have been a valuable study to analyse and draw insights from had it been accessible. Moreover, pieces of grey literature, such as reports by NGOs, could potentially have identified more literature for inclusion in this review. However, these pieces of literature were not explored due to resource constraints.

The second limitation of this study is its ability to provide generalisable conclusions, given the small number of economic evaluations included within the study. Although the number of studies identified is an important reflection of the existing literature in this field, the generalisability of conclusions provided by this review may have benefitted from using less stringent inclusion criteria with regards to the comprehensive nature of the model and the type of economic evaluations included. For instance, cost-benefit studies or papers where the economic evaluation was nested rather than comprising of a stand-alone economic model, could have been considered. That said, the inclusion criteria for this review were selected with the specific purpose of informing future decision models on the natural history of leprosy. Therefore, taking a wider scope would have compromised this study’s ability to fulfil this function.

Recommendations for policy and further research

The main purpose of this review was to contribute to the development of future model-based economic evaluations targeted at preventing and treating patients with leprosy ulcers caused by peripheral neuropathy. In doing so, this study has highlighted the need for more model-based economic evaluations that pertain specifically to leprosy ulcers in LMICs and that these evaluations need to be conducted to a higher methodological standard.

#### Search strategy

Database: Ovid MEDLINE(R) ALL <1946 to April 30, 2020>

Search Strategy:

--------------------------------------------------------------------------------

1 exp Foot Ulcer/ (10136)

2 ((foot or feet or plantar or sole or soles or heel or heels or toe$) adj3 ulcer$).ti,ab. (7540)

3 (diabet$ adj3 (foot or feet)).ti,ab. (9287)

4 ((diabet$ or neuropath$ or trophic) adj3 ulcer$).ti,ab. (7022)

5 1 or 2 or 3 or 4 (16892)

6 exp Leprosy/ (22284)

7 (lepro$ or lepra$ or (Hansen$ adj disease)).ti,ab. (24999)

8 6 or 7 (28227)

9 Buruli Ulcer/ (572)

10 Mycobacterium ulcerans/ (771)

11 (buruli adj3 ulcer$).ti,ab. (988)

12 ((Bairnsdale or Searls or Daintree or Mossman or Kumasi) adj3 ulcer$).ti,ab. (23)

13 (mycobacterium adj (ulcer$ or buruli)).ti,ab. (959)

14 9 or 10 or 11 or 12 or 13 (1354)

15 economics/ (27174)

16 exp "costs and cost analysis"/ (234689)

17 economics, dental/ (1911)

18 exp "economics, hospital"/ (24391)

19 economics, medical/ (9068)

20 economics, nursing/ (3997)

21 economics, pharmaceutical/ (2927)

22 (economic$ or cost$ or price or prices or pricing or pharmacoeconomic$).ti,ab. (816473)

23 (expenditure$ not energy).ti,ab. (29404)

24 value for money.ti,ab. (1666)

25 budget$.ti,ab. (28926)

26 or/15-25 (967369)

27 ((energy or oxygen) adj cost).ti,ab. (4075)

28 (metabolic adj cost).ti,ab. (1393)

29 ((energy or oxygen) adj expenditure).ti,ab. (24774)

30 or/27-29 (29267)

31 26 not 30 (960624)

32 letter.pt. (1073143)

33 editorial.pt. (525826)

34 historical article.pt. (357841)

35 or/32-34 (1937401)

36 31 not 35 (924061)

37 exp animals/ not humans/ (4694664)

38 36 not 37 (857381)

39 5 and 38 (1405)

40 8 and 38 (571)

41 14 and 38 (69)

42 39 or 40 or 41 (2024)

43 limit 42 to yr="2000 -Current" (1629)

44 limit 42 to (english or portuguese) (1838)

45 limit 42 to (yr="2000 -Current" and (english or portuguese)) (1501)

46 ("28976723" or "30337474" or "27489228" or "25767565" or "18400121" or "11298704").ui. (6)

47 45 or 46 (1501)

48 46 not 45 (0)

***************************

1.

Symposium review: Multiple-trait single-step genomic evaluation for hoof health. [Review]

Malchiodi F; Jamrozik J; Christen AM; Fleming A; Kistemaker GJ; Richardson C; Daniel V; Kelton DF; Schenkel FS; Miglior F.

Journal of Dairy Science. 2020 Apr 21.

[Journal Article. Review] [2020/04/26 06:00]

UI: 32331881

Hoof lesions represent an important issue in modern dairy herds, with reported prevalence in different countries ranging from 40 to 70%. This high prevalence of hoof lesions has both economic and social consequences, resulting in increased labor expenses and decreasing animal production, longevity, reproduction, health, and welfare. Therefore, a key goal of dairy herds is to reduce the incidence of hoof lesions, which can be achieved both by improving management practices and through genetic selection. The Canadian dairy industry has recently released a hoof health sub-index. This national genetic evaluation program for hoof health was achieved by creating a centralized data collection system that routinely transfers data recorded by hoof trimmers into a coherent and sustainable national database. The 8 most prevalent lesions (digital dermatitis, interdigital dermatitis, interdigital hyperplasia, heel horn erosion, sole hemorrhage, sole ulcer, toe ulcer, and white line lesion) in Canada are analyzed with a multiple-trait model using a single-step genomic BLUP method. Estimated genomic breeding values for each lesion are combined into a sub-index according to their economic value and prevalence. In addition, data recorded within this system were used to create an interactive management report for dairy producers by Canadian DHI, including the prevalence of lesions on farm, their trends over time, and benchmarks with provincial and national averages.

Copyright © 2020 American Dairy Science Association. Published by Elsevier Inc. All rights reserved.

Abbreviated Source

J Dairy Sci. 2020 Apr 21.

Version ID

1

Record Owner

From MEDLINE, a database of the U.S. National Library of Medicine.

Status

Publisher

Authors Full Name

Malchiodi, F; Jamrozik, J; Christen, A-M; Fleming, A; Kistemaker, G J; Richardson, C; Daniel, V; Kelton, D F; Schenkel, F S; Miglior, F.

Institution

Malchiodi, F. Semex Alliance, Guelph, ON, N1H 6J2, Canada; Centre for Genetic Improvement of Livestock, Department of Animal Biosciences, University of Guelph, Guelph, ON, N1G 1Y2, Canada. Electronic address: fmalchio@uoguelph.ca. Jamrozik, J. Centre for Genetic Improvement of Livestock, Department of Animal Biosciences, University of Guelph, Guelph, ON, N1G 1Y2, Canada; Lactanet Canada, Guelph, ON N1K 1E5, Canada.

Christen, A-M. Lactanet Canada, Sainte-Anne-de-Bellevue, QC H9X 3R4, Canada.

Fleming, A. Lactanet Canada, Guelph, ON N1K 1E5, Canada.

Kistemaker, G J. Lactanet Canada, Guelph, ON N1K 1E5, Canada.

Richardson, C. School of Applied Systems Biology, La Trobe University, Bundoora, Victoria 3086, Australia.

Daniel, V. Vic's Custom Clips, Arva, ON N0M 1C0, Canada.

Kelton, D F. Department of Population Medicine, Ontario Veterinary College, University of Guelph, Guelph, ON N1G 1Y2, Canada.

Schenkel, F S. Centre for Genetic Improvement of Livestock, Department of Animal Biosciences, University of Guelph, Guelph, ON, N1G 1Y2, Canada.

Miglior, F. Centre for Genetic Improvement of Livestock, Department of Animal Biosciences, University of Guelph, Guelph, ON, N1G 1Y2, Canada.

NLM Journal Name

Journal of dairy science

Publishing Model

Journal available in: Print-Electronic. Citation processed from: Internet

NLM Journal Code

hwv, 2985126r

ISO Journal Abbreviation

J. Dairy Sci.

Journal Subset

Index Medicus

Country of Publication

United States

Keyword Heading

hoof lesions hoof trimmer

single-step genomic evaluation.

ISSN Electronic

1525-3198

ISSN Linking

0022-0302

Publisher Item Identifier

S0022-0302(20)30315-5

Digital Object Identifier

<https://dx.doi.org/10.3168/jds.2019-17755>

Article Identifier

S0022-0302(20)30315-5 [pii] 10.3168/jds.2019-17755 [doi]

Publication Status

aheadofprint

Publication History Status

2019/10/15 [received] 2020/01/17 [accepted]

Language

English

Electronic Date of Publication

20200421

Date of Publication

2020 Apr 21

Year of Publication

2020

Revision Date

20200425

Update Date

20200427

### Philips checklist methodological quality assessment

| Quality crtierion |  | Wu et al | Prada et al | Cárde-nas et al |
| --- | --- | --- | --- | --- |
| S1 | Is there a clear statement of the decision problem? | 1 | 1 | 1 |
|  | Is the objective of the evaluation and model specified and consistent with the stated decision problem? | 1 | 1 | 1 |
|  | Is the primary decision-maker specified? | n/a | n/a | n/a |
| S2 | Is the perspective of the model stated clearly? | 1 | 1 | 1 |
|  | Are the model inputs consistent with the stated perspective? | 0 | 1 | 0.5 |
|  | Has the scope of the model been stated and justified? | 1 | 1 | 1 |
|  | Are the outcomes of the model consistent with the perspective, scope and overall objective of the model? | 1 | 1 | 0.5 |
| S3 | Is the structure of the model consistent with a coherent theory of the health condition under evaluation? | 1 | 0.5 | 0.5 |
|  | Are the sources of data used to develop the structure of the model specified? | 0 | 0 | 0 |
|  | Are the causal relationships described by the model structure justified appropriately? | 0.5 | 0.5 | 0.5 |
| S4 | Are the structural assumptions transparent and justified? | 0.5 | 0.5 | 0.5 |
| S5 | Is there a clear definition of the options under evaluation? | 1 | 1 | 1 |
|  | Have all feasible and practical options been evaluated? | 1 | 1 | 1 |
|  | Is there justification for the exclusion of feasible options? | n/a | n/a | n/a |
| S6 | Is the chosen model type appropriate given the decision problem and specified causal relationships within the model? | 1 | 1 | 0.5 |
| S7 | Is the time horizon of the model sufficient to reflect all important differences between options? | 0.5 | 0.5 | 0 |
|  | Are the time horizon of the model, the duration of treatment and the duration of treatment effect described and justified? | 0 | 0.5 | 0 |
| S8 | Do the disease states (state transition model) or the pathways (decision tree model) reflect the underlying biological process of  the disease in question and the impact of interventions? | 1 | 0.5 | 0.5 |
|  | Are the structural assumptions reasonable given the overall objective, perspective and scope of the model? | 1 | 0 | 0.5 |
| S9 | Is the cycle length defined and justified in terms ofthe natural history of disease? | 0 | 0.5 | n/a |
| D1 | Are the data identification methods transparent and appropriate given the objectives of the model? | 0.5 | 0.5 | 0.5 |
|  | Where choices have been made between data sources, are these justified appropriately? | n/a | n/a | n/a |
|  | Has particular attention been paid to identifying data for the important parameters in the model? | 1 | 1 | 1 |
|  | Has the quality of the data been assessed appropriately? | 0 | 0 | 0 |
|  | Where expert opinion has been used, are the methods described and justified? | 0 | 0 | n/a |
| D2 | Is the data modelling methodology based on justifiable statistical and epidemiological techniques? | 0 | 0 | 0 |
| D2A | Is the choice of baseline data described and justified? | 0.5 | 0.5 | 0.5 |
|  | Are transition probabilities calculated appropriately? | n/a | n/a | n/a |
|  | Has a half-cycle correction been applied to both cost and outcome? | 0 | 0 | n/a |
|  | If not, has this omission been justified? | 0 | 0 | n/a |
| D2B | If relative treatment effects have been derived from trial data, have they been synthesised using appropriate techniques? | n/a | n/a | n/a |
|  | Have the methods and assumptions used to extrapolate short-term results to final outcomes been documented and justified? | 0.5 | 0.5 | 0.5 |
|  | Have alternative extrapolation assumptions been explored through sensitivity analysis? | 0 | 0 | 0 |
|  | Have assumptions regarding the continuing effect of treatment once treatment is complete been documented and justified? | 0 | 0 | 0 |
|  | Have alternative assumptions regarding the continuing effect of treatment been explored through sensitivity analysis? | 0 | 0 | 0 |
| D2C | Are the costs incorporated into the model justified? | 1 | 1 | 1 |
|  | Has the source for all costs been described? | 1 | 1 | 1 |
|  | Have discount rates been described and justified given the target decision-maker? | 1 | 1 | 1 |
| D2D | Are the utilities incorporated into the model appropriate? | 1 | 0 | n/a |
|  | Is the source for the utility weights referenced? | 1 | 0 | n/a |
|  | Are the methods of derivation for the utility weights justified? | 0.5 | 0 | n/a |
| D3 | Have all data incorporated into the model been described and referenced in sufficient detail? | 0.5 | 0.5 | 0.5 |
|  | Has the use of mutually inconsistent data been justified (i.e. are assumptions and choices appropriate)? | n/a | n/a | n/a |
|  | Is the process of data incorporation transparent? | 0 | 0.5 | 0 |
|  | If data have been incorporated as distributions, has the choice of distribution for each parameter been described and justified? | 0.5 | 0.5 | 0.5 |
|  | If data have been incorporated as distributions, is it clear that second order uncertainty is reflected? | 1 | 1 | 1 |
| D4 | Have the four principal types of uncertainty been addressed? | 0 | 0 | 0 |
|  | If not, has the omission of particular forms of uncertainty been justified? | 0 | 0 | 0 |
| D4A | Have methodological uncertainties been addressed by running alternative versions of the model with different methodological assumptions? | 0 | 0 | 0 |
| D4b | Is there evidence that structural uncertainties have been addressed via sensitivity analysis? | 0 | 0 | 0 |
| D4c | Has heterogeneity been dealt with by running the model separately for different subgroups? | 0 | 0 | 0 |
| D4d | Are the methods of assessment of parameter uncertainty appropriate? | 1 | 1 | 1 |
| C1 | If data are incorporated as point estimates, are the ranges used for sensitivity analysis stated clearly and justified? | 0.5 | 0.5 | 0.5 |
|  | Is there evidence that the mathematical logic of the model has been tested thoroughly before use? | 0 | 0 | 0 |
| C2 | Are any counterintuitive results from the model explained and justified? | n/a | n/a | n/a |
|  | If the model has been calibrated against independent data, have any differences been explained and justified? | n/a | n/a | n/a |
|  | Have the results of the model been compared with those of previous models and any differences in results explained? | 0.5 | 0.5 | 0.5 |
|  |  | 50% | 46% | 46% |

### CHEERs checklist quality assessment

|  |  | Wu et al | | Prada et al | | Cárdenas et al | |
| --- | --- | --- | --- | --- | --- | --- | --- |
|  |  | Score | Page | Score | Page | Score | Page |
| Title and abstract | 1) Title | 1 | p.320 | 1 | p.1 | 1 | p.1 |
|  | 2) Abstract | 1 | p.320 | 1 | p.1 | 1 | p.1 |
| Introduction | 3) Background and Objectives | 1 | p.320-321 | 1 | p.2 | 1 | p.2 |
| Methods | 4) Target Population and Subgroups | 0.5 | p.321 | 0.5 | p.2 | 1 | p.2 |
|  | 5) Setting and Location | 1 | p.321 | 1 | p.2 | 1 | p.2 |
|  | 6) Study Perspective | 0.5 | p.321,323 | 1 | p.3 | 1 | p.4 |
|  | 7) Comparators | 1 | p.321 | 0.5 | p.2 | 1 | p.2 |
|  | 8) Time horizon | 0.5 | p.325 | 1 | p.2 | 0.5 | p.2 |
|  | 9) Discount rate | 1 | p.321 | 1 | p.3 | 1 | p.5 |
|  | 10) Choice of Health Outcomes | 0.5 | p.321 | 0.5 | p.2 | 0.5 | p.1 |
|  | 11a) Measurement of effectiveness | n/a |  | n/a |  | n/a |  |
|  | 11b) "" | 1 | p.321-323 | 0 |  | 0.5 | p.2-4 |
|  | 12) Measurement and valuation of preference-based outcomes | 1 | p.322, 323 | 0 |  | n/a |  |
|  | 13a) Estimating resources and costs | n/a |  | n/a |  | n/a |  |
|  | 13b)"" | 1 | p.323 | 1 | p.3-4 | 1 | p.4 |
|  | 14) Currency, price date, and conversion | 1 | p.321 | 1 | p.3-4 | 0.5 | p.4 |
|  | 15) Choice of Model | 0.5 | p.321 | 1 | p.2-3 | 0.5 | p.2 |
|  | 16) Assumptions | 1 | p.321,323 | 0.5 | p.3 | 1 | p.4 |
|  | 17) Analytic Methods | 1 | p.323 | 1 | p.4 | 1 | p.5 |
| Results | 18) Study Parameters | 1 | p.323 | 1 | p.3-4 | 1 | p.3,5 |
|  | 19) Incremental costs and outcomes | 1 | p.323 | 1 | p.4 | 1 | p.6 |
|  | 20a) Characterizing uncertainty | n/a |  | n/a |  | n/a |  |
|  | 20b) "" | 1 | p.324-325 | 1 | p.4-5 | 1 | p.6-7 |
|  | 21) Characterizing heterogeneity | 0 |  | 0 |  | 0 |  |
| Discussion | 22) Study findings, limitations, generalizability, and current knowledge | 1 | p.324-326 | 1 | p.5-6 | 1 | p.8 |
| Other | 23) Source of Funding | 1 | p.326 | 0.5 | p.6 | 1 | p.9 |
|  | 24) Conflicts of Interest | 1 | p.326 | 0 |  | 0 |  |
| Score |  | 85% |  | 73% |  | 80% |  |

### Appendix B. Literature searches to inform decision model parameters

Three targeted searches were carried out for each of the parameters or groups of parameters required: clinical parameters, health-related quality of life (HRQoL) and costs and resource use. References from the original NIHR bid and the study protocols were also reviewed. All three searches used as inclusion criteria studies which 1) included patients with leprosy ulcers (although studies including a proportion of diabetic ulcer patients were acceptable), 2) were based in an LMIC; 3) were published from 2000; 4) were of sufficient sample size; 5) were in English.

#### Search 1: Clinical parameters

A search of Ovid MEDLINE was created by a researcher at the Centre for Reviews and Dissemination. Searches were tested for the inclusion of papers known to be relevant to the project team. The search strategy was designed to identify studies which included the following parameters:

- Risk or rate of healing of leprosy ulcers when treated with standard care
- Risk of recurrence of the same leprosy ulcer
- Risk of new leprosy ulcer
- Risk of developing a complicated ulcer

Appendix B Figure 1 shows the PRISMA diagram of the search for clinical parameters. It was carried out on 7th July 2022 and identified 560 records after the removal of duplicates. 509 were excluded at the title and abstract screen and full papers reviewed for 65 studies. On full text screen 57 studies were excluded (See Appendix B Figure 1). A number of the studies included non-healing plantar ulcers that were due to causes other than leprosy (for example, diabetes, lumbosacral meningomyelocele, trauma) alongside the leprosy ulcers. As long as the study included at least some leprosy patients it was included. One reviewer (NG) screened titles and abstracts. The full papers were screened in the first instance by one reviewer (NG) and then rescreened by another two reviewers (JO and MS). Input was then gathered from project partners to inform the final choice of clinical model parameters.

##### Appendix B Figure 1: Prisma of literature search for clinical parameters to inform the model


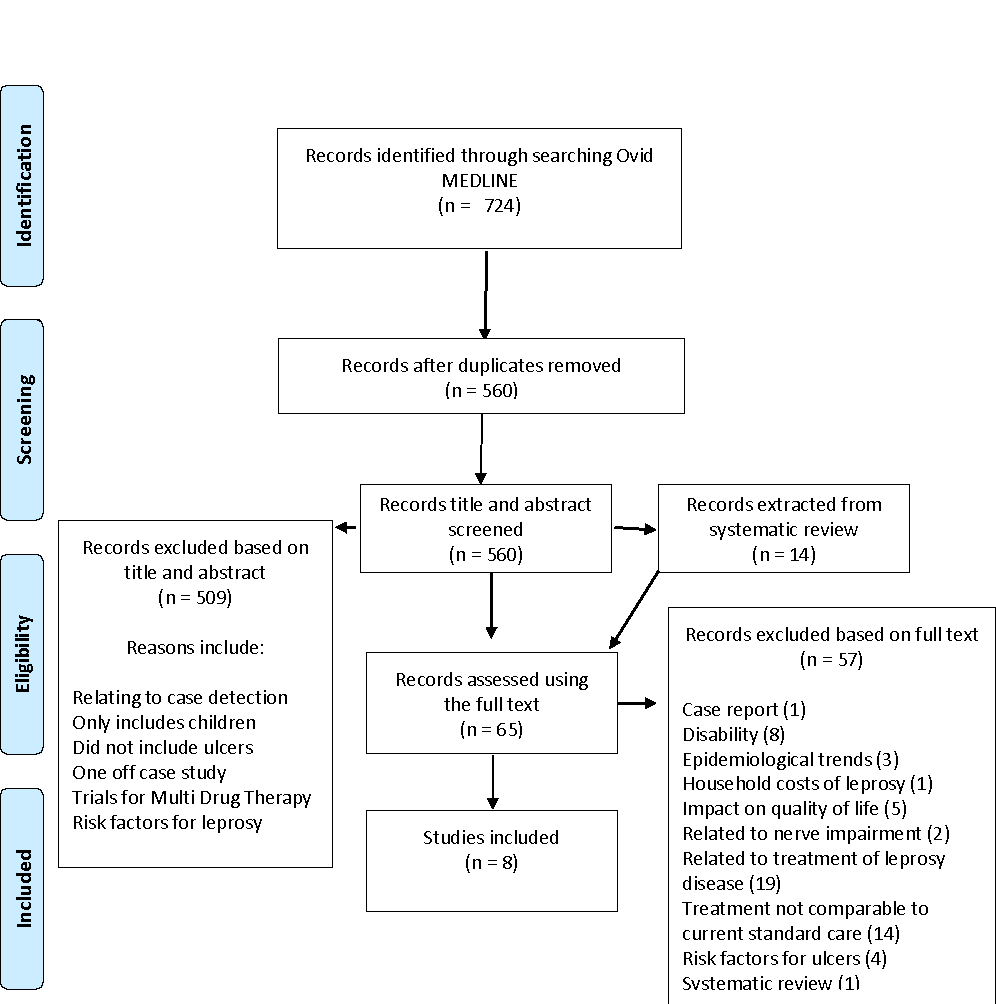


Amongst the papers there was a systematic review of interventions to treat ulcers caused by leprosy which included 14 studies. All these studies reported ulcer healing but most often it was reduction in ulcer area or volume up to the end of the trial. In some instances, the trials were short, such as 4 weeks with no follow up. There were three RCTs from this review which included saline or conventional dressing therapy (the standard of care for leprosy ulcers); however, one was published in 1993, so was excluded, and another did not give any detail of the conventional dressing treatment.

Included studies were extracted and are summarised (Appendix B Table 1). No studies were identified that could inform healing in the absence of treatment. Treatments included “standard of care” and Platelet Rich Plasma or Fibrin dressings.

##### Appendix B Table 1: Ulcer healing

| Author (year) | Study type | Sample size | Treatment | Follow up | Mean time to healing or rate of healing | Percentage unhealed |
| --- | --- | --- | --- | --- | --- | --- |
| Standard care studies identified | | | | | | |
| Rai et al. (2016) | Prospective cohort study | 30 (17 leprosy) | Dressing changes every two weeks. Walking aids to reduce pressure. | 8 weeks | 6.73 weeks (SD 1.92) | 20% |
| Saraf et al. (2000) | Prospective cohort study | 75 (37 leprosy patients) | Treated every two weeks. Details not reported. | Not reported | 8.05 weeks (SD 3.24) leprosy only | 0%  All patients followed to complete healing |
| (Bhatia et al., 2004) | Randomised Control Trial | 50 (15 received the saline dressing treatment) | Daily wound dressing. Patients treated in hospital. | 4 weeks | 49.1% (18.2 SD) reduction in ulcer area by 4 weeks. | 100% |
| (Barreto and Salgado, 2010) | Randomised Control Trial | 25 patients (12 received standard care) | Daily wound dressings, orientation of use of adapted footwear. | 13 weeks | 0.82cm2 (0.00 to 1.66 CI) reduction in ulcer area at 13 weeks | 100% |
| Non-standard care studies identified | | | | | | |
| Anandan et al. (2016) | Prospective cohort study | 50 | Platelet Rich Plasma treatment at weekly dressing change. Walking aids to reduce pressure. | 3 months | 4.38 weeks (SD NR) | 8% |
| (Ghatge et al., 2021) | Prospective cohort study | 25 | Weekly PRF treatment applied to the wound. | 6 weeks | 5 weeks (SD 1.32) for those that healed.  90% reduction in ulcer volume across all patients | 32% |

Two papers were extracted that could inform the probability of recurrence following an ulcer being healed. A number of studies reported the number of cases presenting that were recurrent ulcers versus new ulcers; however, these cannot inform the probability that a healed ulcer will recur. Only two studies reported this (Appendix B Table 2).

##### Appendix B Table 2: Ulcer recurrence

| Author (year) | Study type | Sample size | Treatment | Follow up | Recurrence rate |
| --- | --- | --- | --- | --- | --- |
| Gahalaut et al. (2005) | Prospective cohort study | 40 | Three different types of surgery | 3 years | 25%  6/10 recurred within 6 months to 1 year |
| Yan et al. (2003) | Prospective cohort study | 1804 | Wide range of treatments including: skin grafting, protective footwear, rest, wound dressing, debridement. | 3 years | 18%  (this was the average across 12 provinces, one province reported 46% recurrence) |

We calculated baseline probability of recurrence from the TABLE trial.

The risk of developing a new leprosy ulcer was taken from Govindasamy et al (2023), which was identified after the review, as K Govindasamy is a member of the project team (11).

#### Search 2: Health-related quality of life

Estimates of HRQoL are drawn from data from the TABLE trial. The face validity of these estimates was assessed with findings from our pragmatic targeted review of the literature. We started with a full text review of all papers relating to HRQoL that came out of the systematic search for clinical parameters. In addition, estimates from WHO were considered and all three studies arising from a review of cost-effectiveness studies for interventions to prevent disability arising from leprosy.

The TABLE study collected health related quality of life data using the EQ-5D alongside the trial. This provides some of the parameters needed for the model. We conducted a targeted and pragmatic literature review to check EQ-5D trial results for face validity. No studies were found which included health related quality of life specifically for patients with ulcers caused by leprosy, however, papers with HRQoL values for leprosy patients more broadly may be indicative.

A systematic review of the cost-effectiveness of intervention to prevent disability in leprosy found three studies (6). Two of these studies included quality of life measures, one used a 20-question survey derived from WHO QOL (7) and the other used DALY weightings (9).

Ravi et al. (2004) were comparing hospitalisation versus ambulatory care in the management of neuritis (involving steroids and a two-week period of rest) occurring in lepra reaction at a hospital in India. The questionnaire had five domains (physical, psychological, level of independence, environment, and social), each question had a maximum score of 5 except the question on pain in the physical domain which had a maximum of 6. Total maximum was 106, with a higher score indicating higher quality of life. Only 17 patients reported QoL, the mean pre-treatment scores in the ambulatory and inpatient group were 61.3 and 68.0, respectively. The mean post treatment scores in both groups were 77.7 and 78.8, respectively.

Remme et al. (2021) undertook a modelling study across four tropical diseases, including leprosy. They used a WHO Disability Adjusted Life Year (DALY) weight of 0.152 for disabling leprosy, a category that encompasses leprosy patients who may be very different in their condition and who require a wide range of treatments. For example, the study simulates cost effectiveness for interventions to cure leprosy, to treat reactions and ulcers, for those needing footwear and self-care education and for those needing reconstructive surgery. They have a weight of 0 for leprosy patients without a disability.

The Institute for Health Metrics and Evaluation (iHME) produce DALY weights. These weights represent the magnitude of health loss associated with a condition on a scale from 0 – 1, with 0 equal to a state of full health and 1 equivalent to death. They provide two values depending on the level of disfigurement (Table 4).

##### Appendix B Table 3: iHME 2019 DALY weights for disfigurement due to leprosy

| Health state | Description | Mean Disability Weight (CI) |
| --- | --- | --- |
| Disfigurement level 1 due to leprosy | Has a slight, visible physical deformity that others notice, which causes some worry and discomfort. | 0.011 (0.005-0.021) |
| Disfigurement level 2 due to leprosy | Has a visible physical deformity that causes others to stare and comment. As a result, the person is worried and has trouble sleeping and concentrating. | 0.067 (0.044-0.096) |

Research by Nanjan Chandran et al. (2021) estimated that the WHO disability weights are too low. In their systematic review they use individual patient data sourced from 667 individuals to estimate leprosy disability weights by grade^^[[1]](#footnote-1)^^ of disability. The overall estimated disability weight for grade 2 disability was 0.26 (95% CI: 0.18–0.34). For grade 1 disability the estimated weight was 0.19 (95%CI: 0.13–0.26) and for grade 0 disability it was 0.13 (95%CI: 0.06–0.19). The disability weight for grade 2 leprosy disability is four times higher than the published GBD 2017 weights and the grade 1 disability weight is nearly twenty times higher.

The iHME provide weights for a decubitus ulcer (pressure ulcer or bedsore) which has a very similar description in terms of visibility and resulting worry but with the added dimension of itching and soreness. They list three levels (mild/moderate/severe) with corresponding weights (0.027/0.188/0.576). (**mild** - has a slight, visible physical deformity that is sometimes sore or itchy. Others notice the deformity, which causes some worry and discomfort; **moderate** - has a visible physical deformity that is sore and itchy. Other people stare and comment, which causes the person to worry. The person has trouble sleeping and concentrating; **severe** - has an obvious physical deformity that is very painful and itchy. The physical deformity makes others uncomfortable, which causes the person to avoid social contact, feel worried, sleep poorly, and think about suicide.)

#### Search 3: Cost and resources use

Costs and resource use for our model are informed by the TABLE trial. However only limited data was collected routinely on resource use, namely: days in hospital prior to discharge and in total by six months, number of visits to any healthcare facility from discharge to 6 months, and time taken at the twice weekly dressing changes. We will need to add to this data using local clinical experts and the academic literature.

A systematic review of the cost-effectiveness of intervention to prevent disability in leprosy found three studies (6). Ravi et al. (2004), whose study was based in India and compared ambulatory versus in-patient management of neuritis in Leprosy. They included direct costs, incurred by the health sector and the patient which included medication, tests and hospitalisation. Direct non-medical costs included transport and food. Indirect costs include wages lost due to illness.

Remme et al. (2021) assuming a 90% success rate estimate costs of US$7 for patients needing treatment for reactions and ulcers, US$75 for those needing footwear and self-care education and US$110 for those needing reconstructive surgery. They mention but do not include reduced household income due to difficulty of obtaining employment and the significant social impact due to stigma.

The third study in the systematic review was an RCT in rural Ethiopia designed to compare canvas shoes with moulded sandals (Seboka and Saunderson, 1996). The only cost included in the study was that of the shoes/sandals.

##### Appendix B. Search 1: Clinical parameters search strategy

Date of search: 7th July 2022

Records retrieved: 560

Database: Ovid MEDLINE(R) ALL <1946 to July 06, 2022>

Search Strategy:

--------------------------------------------------------------------------------

1 exp Leprosy/ (23035)

2 (lepro$ or lepra$ or (Hansen$ adj disease)).ti. (22337)

3 1 or 2 (26238)

4 Ulcer/ (14923)

5 skin ulcer/ or leg ulcer/ or foot ulcer/ (19702)

6 (ulcer$ or wound$).ti. (178986)

7 (lesion$ or nodule$ or macule$ or papule$).ti,ab. (999868)

8 (skin or tissue$ or cutaneous).mp. (3205320)

9 7 and 8 (262642)

10 4 or 5 or 6 or 9 (449701)

11 3 and 10 (2099)

12 deform$.ti,ab. (160686)

13 disabilit$.ti,ab. (219909)

14 12 or 13 (377411)

15 3 and 14 (1389)

16 risk/ (127770)

17 exp Risk Assessment/ (304462)

18 risk factors/ (927071)

19 Probability/ (59349)

20 Epidemiologic Studies/ (9132)

21 Survival Analysis/ (145011)

22 Proportional Hazards Models/ (88616)

23 risk.ti,ab. (2499789)

24 probabilit$.ti,ab. (240058)

25 (epidemiolog$ or clinicoepidemiolog$).ti,ab. (423227)

26 (survival adj2 (analys$ or curve$ or rate$1 or status or function)).ti,ab. (252264)

27 (proportional hazard$ adj2 (model$ or analys$ or regression)).ti,ab. (77780)

28 16 or 17 or 18 or 19 or 20 or 21 or 22 or 23 or 24 or 25 or 26 or 27 (3698315)

29 11 and 28 (179)

30 15 and 28 (369)

31 29 or 30 (504)

32 exp animals/ not humans/ (5023939)

33 31 not 32 (497)

34 limit 33 to english language (449)

35 limit 34 to yr="2000 -Current" (363)

36 exp Leprosy/ (23035)

37 (lepro$ or lepra$ or (Hansen$ adj disease)).ti. (22337)

38 36 or 37 (26238)

39 Ulcer/ (14923)

40 skin ulcer/ or leg ulcer/ or foot ulcer/ (19702)

41 (ulcer$ or wound$).ti. (178986)

42 (lesion$ or nodule$ or macule$ or papule$).ti,ab. (999868)

43 (skin or tissue$ or cutaneous).mp. (3205320)

44 42 and 43 (262642)

45 39 or 40 or 41 or 44 (449701)

46 38 and 45 (2099)

47 Recurrence/ (195320)

48 Reinfection/ (677)

49 symptom flare up/ (1082)

50 Remission Induction/ (43853)

51 Remission, Spontaneous/ (16934)

52 (recur$ or re-cur$).ti,ab. (670518)

53 (reoccur$ or re-occur$).ti,ab. (3912)

54 (reinfect$ or re-infect$).ti,ab. (13902)

55 (reactivat$ or re-activat$).ti,ab. (48104)

56 (relaps$ or re-laps$).ti,ab. (207265)

57 (remission$ or re-mission).ti,ab. (138813)

58 (refractor$ or re-fractor$).ti,ab. (150268)

59 (recrudesce$ or re-crudesce$).ti,ab. (3736)

60 (reappear$ or re-appear$ or reemerge$ or re-emerge$).ti,ab. (19810)

61 47 or 48 or 49 or 50 or 51 or 52 or 53 or 54 or 55 or 56 or 57 or 58 or 59 or 60 (1220709)

62 46 and 61 (215)

63 exp animals/ not humans/ (5023939)

64 62 not 63 (210)

65 limit 64 to english language (185)

66 limit 65 to yr="2000 -Current" (122)

67 exp Leprosy/ (23035)

68 (lepro$ or lepra$ or (Hansen$ adj disease)).ti. (22337)

69 67 or 68 (26238)

70 Ulcer/ (14923)

71 skin ulcer/ or leg ulcer/ or foot ulcer/ (19702)

72 (ulcer$ or wound$).ti. (178986)

73 (lesion$ or nodule$ or macule$ or papule$).ti,ab. (999868)

74 (skin or tissue$ or cutaneous).mp. (3205320)

75 73 and 74 (262642)

76 70 or 71 or 72 or 75 (449701)

77 69 and 76 (2099)

78 Wound Healing/ (102848)

79 (heal$ or nonheal$ or non-heal$).ti,ab. (3476162)

80 78 or 79 (3515388)

81 77 and 80 (349)

82 risk/ (127770)

83 exp Risk Assessment/ (304462)

84 risk factors/ (927071)

85 Probability/ (59349)

86 Epidemiologic Studies/ (9132)

87 Survival Analysis/ (145011)

88 Proportional Hazards Models/ (88616)

89 risk.ti,ab. (2499789)

90 probabilit$.ti,ab. (240058)

91 (epidemiolog$ or clinicoepidemiolog$).ti,ab. (423227)

92 (survival adj2 (analys$ or curve$ or rate$1 or probabilit$ or status or function)).ti,ab. (260467)

93 (proportional hazard$ adj2 (model$ or analys$ or regression)).ti,ab. (77780)

94 82 or 83 or 84 or 85 or 86 or 87 or 88 or 89 or 90 or 91 or 92 or 93 (3698315)

95 81 and 94 (55)

96 ((heal$ or cure$) adj4 (rate$ or speed or duration or time or fast$ or slow$ or delay$ or prolong$ or long$ or short$)).ti,ab. (176948)

97 77 and 96 (41)

98 95 or 97 (91)

99 exp animals/ not humans/ (5023939)

100 98 not 99 (89)

101 limit 100 to english language (86)

102 limit 101 to yr="2000 -Current" (72)

103 exp Leprosy/co [Complications] (3241)

104 risk/ (127770)

105 exp Risk Assessment/ (304462)

106 risk factors/ (927071)

107 Probability/ (59349)

108 Epidemiologic Studies/ (9132)

109 Survival Analysis/ (145011)

110 Proportional Hazards Models/ (88616)

111 risk.ti,ab. (2499789)

112 probabilit$.ti,ab. (240058)

113 (epidemiolog$ or clinicoepidemiolog$).ti,ab. (423227)

114 (survival adj2 (analys$ or curve$ or rate$1 or probabilit$ or status or function)).ti,ab. (260467)

115 (proportional hazard$ adj2 (model$ or analys$ or regression)).ti,ab. (77780)

116 104 or 105 or 106 or 107 or 108 or 109 or 110 or 111 or 112 or 113 or 114 or 115 (3698315)

117 103 and 116 (288)

118 exp animals/ not humans/ (5023939)

119 117 not 118 (288)

120 limit 119 to english language (265)

121 limit 120 to yr="2000 -Current" (167)

122 35 or 66 or 102 or 121 (560)

### Appendix C. Time to healing

#### Data

The trial included 130 patients and baseline characteristics are reported in full in Napit et al, 2024 (12). The mean age of participants was 54 years and mean ulcer area (measured in cm^2^) was 3.8. Four patients withdrew from the study for reasons unrelated to their ulcer, and were censored. Their available data is therefor used up until the point they leave the study, after which their outcome is no longer observed or considered in the analysis.

#### Assessment of model fit

We first assessed time to healing using Cox Proportional Hazards at 42 days, as the clinical trial paper reported this as their main analysis. The Cox Proportional Hazards at 42 days adjusted for trial ulcer size and baseline age are 1.3 (0.8 - 2.0). The Kaplan-Meier Survival curve for 42 days is shown in Appendix C Figure1 below and matches the trial paper (12).

##### Appendix C Figure 1. Kaplan-Meier Survival Curve


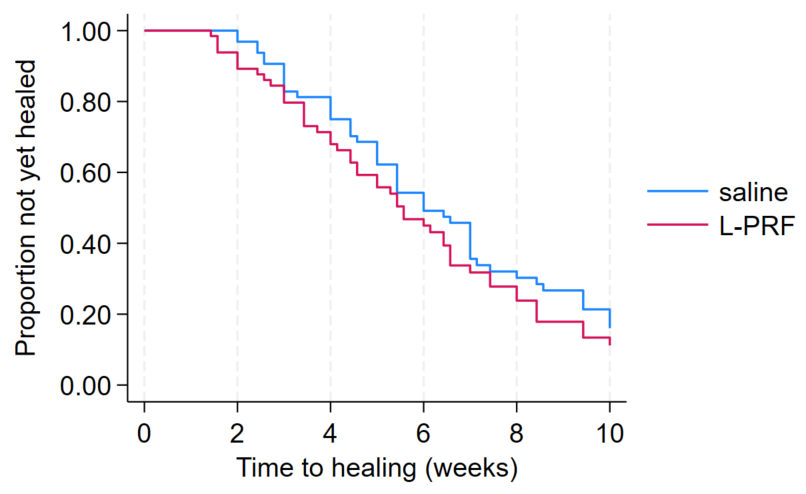


A fully parametric model was required to model time to ulcer healing and extrapolate beyond the timeline of the clinical trial. In order to better extrapolate time to healing beyond the study time horizon and model time to event, we make use of the full dataset collected where patients were followed up for up to 70 days. After 42 days, patients were allowed to switch trial arms, and seven did so. An intention-to-treat analysis was therefore performed whereby all patients were analysed according to their originally assigned treatment groups, regardless of any post-randomization arm switches. This approach ensures that the analysis reflects a conservative estimate of treatment effect, minimising bias due to crossover and preserving the randomised trial structure. Using the full dataset means fewer patients were censored providing a more accurate picture of the true distribution of healing times.

Cox-Snell residuals were used to assess the goodness of fit of the models under consideration (see Appendix C Figure 2). Weibull, loglogistic, lognormal and generalised gamma all appeared to fit reasonably well.

##### Appendix C Figure 2. Cox-Snell residuals


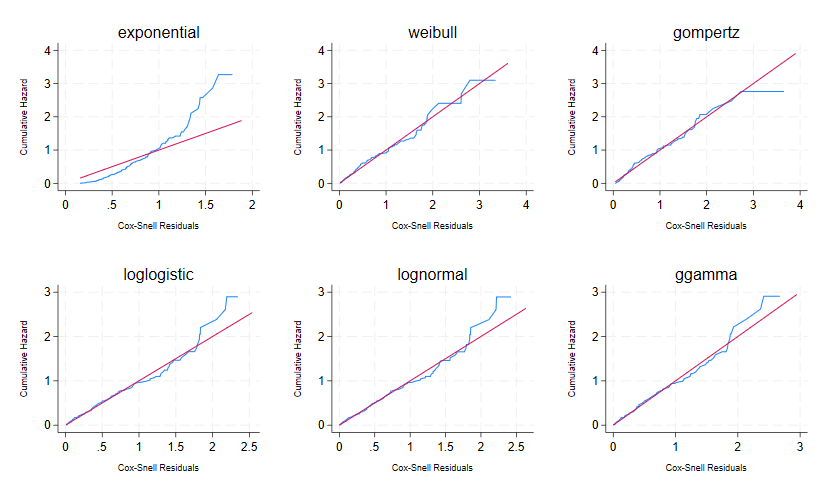


We then assessed model fit using Akaike information criterion, Bayesian information criterion both without and with additional covariates (age and ulcer area (in cm^2) at baseline following the clinical trial).

##### Appendix C Table 1. Estimated coefficients, Akaike information criterion, and Bayesian information criterion

|  | Coefficient | AIC | BIC |
| --- | --- | --- | --- |
| Without additional covariates | | | |
| Weibull | -0.107 | 231 | 239 |
| Exponential | -0.121 | 294 | 300 |
| Log Logistic | -0.131 | 229 | 238 |
| Lognormal | -0.150 | 227 | 235 |
| Generalised gamma | -0.138 | 228 | 240 |
| With baseline age and ulcer size covariates | | | |
| Exponential | -0.144 | 288 | 300 |
| Weibull | -0.135 | 215 | 229 |
| Log Logistic | -0.157 | 216 | 230 |
| Lognormal | -0.160 | 213 | 227 |
| Generalised gamma | -0.150 | 214 | 231 |

Appendix C Figure 3 shows survival truncated at 52 weeks. The vertical black dashed line has been inserted at 6 months as a marker for comparability when visually assessing the models. The y-axis represents survival probability, where at cycle zero everyone in the trial has an ulcer. The survival curve shows the estimated proportion of patients who still have an ulcer at each time point (weeks). The dashed blue line represents the standard of care (Saline) trial arm and the red line represents the treatment (L-PRF) trial arm. The probability of healing differs by week, based on the estimated survival curves (see Appendix C for equations) with patients healing more quickly with L-PRF than Saline.

##### Appendix C Figure 3. Modelled survival truncated at 52 weeks


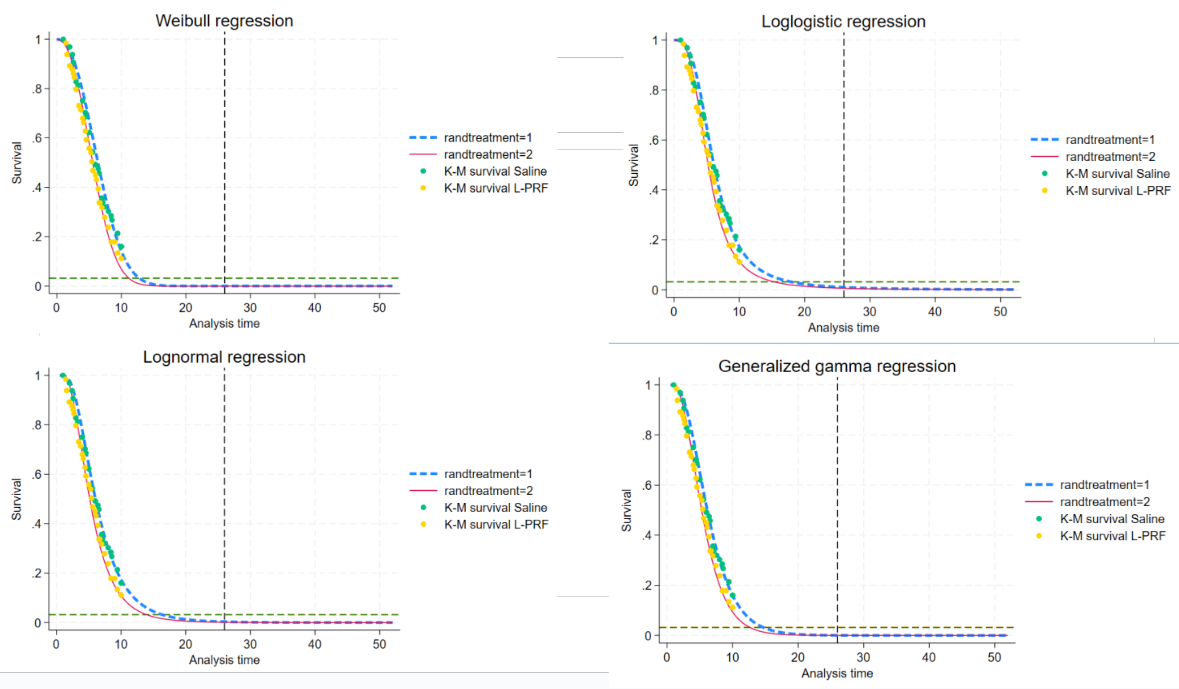


Randtreatment = 1 refers to Saline and randtreatment = 2 refers to L-PRF.

We chose the Weibull model with ulcer size and age at baseline as our main analysis, and report the estimation output, equations and Cholesky decomposition matrix for all four models in the sections that follow. This matrix facilitates the generation of correlated random variables, capturing the joint uncertainty in parameter estimates for the probabilistic sensitivity analysis. Applying the Cholesky decomposition accounts for correlations between model parameters, ensuring that the random draws in the Monte Carlo simulations in the probabilistic sensitivity analysis reflect the interdependencies observed in the estimated parameters.

#### Weibull model

The estimated model coefficients, standard errors, 95% confidence intervals, Hazard ratios (where appropriate) and model diagnostics for the Weibull model are reported in Appendix C Table 2 below. The Weibull shape parameter (p) for our model was estimated to be 2.3 (95% CI: 2.0–2.7), suggesting an increasing hazard over time.

##### Appendix C Table 2. Weibull Proportional Hazards Model for Time to Healing in Patients with Plantar Ulcers

| **Variable** | **Coefficient** | **Standard error** | **95% confidence intervals** | | **Hazard ratio** |
| --- | --- | --- | --- | --- | --- |
| Constant | $\beta_{0}$=-3.4390 | 0.5542 | -4.5253 | -2.353 | 0.0321 |
| Age | $\beta_{1}$=-0.0228 | 0.0063 | -0.0351 | -0.0106 | 0.9774 |
| Ulcer area | $\beta_{2}=$-0.0754 | 0.0381 | -0.1500 | -0.0008 | 0.9274 |
| L-PRF | $\beta_{3}=$0.3124 | 0.2030 | -0.0855 | 0.7104 | 1.3668 |
| **Model statistics** |  |  |  |  |  |
| Shape parameter (p) | 2.3113 | 0.1859 | 1.9742 | 2.7060 |  |
| ln_p | 0 .8378 | 0.0804 | 0.6802 | 0.9955 |  |
| Log likelihood | -102.49923 |  |  |  |  |
| LR chi-squared | 19.84 |  |  |  |  |
| Prob > chi-squared | 0.0002 |  |  |  |  |
| Number of observations | 129 |  |  |  |  |

The time dependent transition probability for healing under standard of care is calculated for each weekly cycle where $LPRF$ equals zero if the patient was treated with standard of care and one if the patient was treated with L-PRF as:

$$tp(t)=1-e^{RR*\lambda*(({t-1)}^{p}-t^{p})}$$

Where $t$ is the cycle, $p$ is estimated in the regression output above and $RR$ and $\lambda$ are calculated as follows using estimates from the regression output:

$$\lambda=e^{{\beta_{0}}+\beta_{1}.Age+\beta_{2}.Ulcer area}$$

$RR$=$e^{\beta_{3}.LPRF}$

Appendix C Table 2 presents the Cholesky decomposition matrix used to characterise the covariance structure of the parameter estimates in the Weibull proportional hazards model.

##### Appendix C Table 2. Cholesky Decomposition Matrix for Covariance Structure in the Weibull Proportional Hazards Model

|  | L-PRF | Age | Ulcer area | Constant | Log(p) |
| --- | --- | --- | --- | --- | --- |
| L-PRF | 0.2030 |  |  |  |  |
| Age | -0.0009 | 0.0062 |  |  |  |
| Ulcer area | 0.0014 | -0.0014 | 0.0380 |  |  |
| Constant | -0.2970 | -0.2551 | -0.1181 | 0.3741 |  |
| Log(p) | 0.0082 | -0.0137 | -0.0051 | -0.0758 | 0.0210 |

#### Log-logistic model

Appendix C Table 3 reports the results of the Log-logistic accelerated failure time model for time to healing.

##### Appendix C Table 3. Log-logistic Accelerated Failure Time Model for Time to Healing in Patients with Plantar Ulcers

| **Variable** | **Coefficient** | **Standard error** | **95% confidence intervals** | | **Hazard ratio** |
| --- | --- | --- | --- | --- | --- |
| Constant | $\beta_{0}$= -1.3054 | 0.2334 | 0.8479 | 1.7628 | 3.6892 |
| Age | $\beta_{1}$= 0.0087 | 0.0032 | 0.0024 | 0.0150 | 1.0088 |
| Ulcer area | $\beta_{2}=$ 0 .0490 | 0.0177 | 0.0144 | 0.0836 | 1.0502 |
| L-PRF | $\beta_{3}=$ 0-.1570 | 0.1011 | -0.3551 | 0.7104 | 0.8547 |
| **Model statistics** |  |  |  |  |  |
| Shape parameter ($\gamma$) | $\gamma$ = 0.3157 | 0.0259 | 0.2687 | 0.3707 |  |
| Log($\gamma$) | -1.1531 | 0.0820 | -1.3138 | -0.9923 |  |
| Log likelihood | -103.0335 |  |  |  |  |
| LR chi-squared | 16.88 |  |  |  |  |
| Prob > chi-squared | 0.0007 |  |  |  |  |
| Number of observations | 129 |  |  |  |  |

The time dependent transition probability for healing $tp(t)$ in each cycle draws upon the survivor function for each cycle $S(t)^{t}$.

$$tp(t)=1-S(t)^{t}/S(t)^{t-1}$$

The survivor function for each cycle $S(t)^{t}$

$$S(t)^{t}=1/(1+(t/\lambda{{*AFT)}^{1/\gamma}})$$

where gamma is the estimated shape parameter in the Log Logistic accelerated failure time model for time to healing where lambda and the accelerated time failure rate for healing are calculated as follows:

$$\lambda=e^{{\beta_{0}}+\beta_{1}.Age+\beta_{2}.Ulcer area}$$

$$AFT=e^{\beta_{3}.LPRF}$$

$LPRF$ equals zero if the patient was treated with standard of care and one if the patient was treated with L-PRF.

Appendix C Table 4 presents the Cholesky decomposition matrix used to characterise the covariance structure of the parameter estimates in the Log-logistic accelerated time failure model.

##### Appendix C Table 4. Cholesky Decomposition Matrix for Covariance Structure in the Log-logistic Accelerated Time Failure Model

|  | L-PRF | Age | Ulcer area | Constant | Log($\gamma$) |
| --- | --- | --- | --- | --- | --- |
| L-PRF | 0.0102 |  |  |  |  |
| Age | 0.0000 | 0.0000 |  |  |  |
| Ulcer area | 0.0001 | 0.0000 | 0.0003 |  |  |
| Constant | -0.0140 | -0.0005 | -0.0013 | 0.0545 |  |
| Log($\gamma$) | 0.0001 | 0.0000 | 0.0000 | -0.0005 | 0.0067 |

#### Lognormal model

Appendix C Table 5 reports the results of the Lognormal accelerated failure time model for time to healing.

##### Appendix C Table 5. Lognormal Accelerated Failure Time Model for Time to Healing in Patients with Plantar Ulcers

| **Variable** | **Coefficient** | **Standard error** | **95% confidence intervals** | | **Hazard ratio** |
| --- | --- | --- | --- | --- | --- |
| Constant | $\beta_{0}$= 1.3020 | 0.2352 | 0.8411 | 1.7629 | 3.6766 |
| Age | $\beta_{1}$= 0.0084 | 0.0031 | 0.0023 | 0.0146 | 1.0085 |
| Ulcer area | $\beta_{2}=$ 0.0523 | 0.0175 | 0.0180 | 0.0867 | 1.0537 |
| L-PRF | $\beta_{3}=$ -0.1598 | 0.0991 | -0.3541 | 0.0345 | 0.8523 |
| **Model statistics** |  |  |  |  |  |
| Shape parameter ($\sigma$) | $\sigma$ = 0.5375 | 0.0389 | 0.4665 | 0.6195 |  |
| Log($\sigma$) | -0.6207 | 0.0723 | -0.7625 | -0.4789 |  |
| Log likelihood | -101.4121 |  |  |  |  |
| LR chi-squared | 18.05 |  |  |  |  |
| Prob > chi-squared | 0.0004 |  |  |  |  |
| Number of observations | 129 |  |  |  |  |

The time dependent transition probability for healing $tp(t)$ in each cycle draws upon the survivor function for each cycle $S(t)^{t}$.

$$tp(t)=1-S(t)^{t}/S(t)^{t-1}$$

The survivor function for each cycle $S(t)^{t}$

$$S(t)^{t}= 1 - \Phi((ln(t)) - (ln(\lambda)+ln(AFT))/\sigma)$$

where Φ denotes a cumulative distribution function (cumulative standard normal). $\sigma$ is the estimated shape parameter in the Lognormal accelerated failure time model for time to healing. Lambda is calculated as follows:

$$\lambda=e^{{\beta_{0}}+\beta_{1}.Age+\beta_{2}.Ulcer area}$$

$$AFT=e^{\beta_{3}.LPRF}$$

$LPRF$ equals zero if the patient was treated with standard of care and one if the patient was treated with L-PRF.

Appendix C Table 4 presents the Cholesky decomposition matrix used to characterise the covariance structure of the parameter estimates in the Log-logistic accelerated time failure model.

##### Appendix C Table 6. Cholesky Decomposition Matrix for Covariance Structure in the Lognormal Accelerated Time Failure Model

|  | L-PRF | Age | Ulcer area | Constant | Log($\gamma$) |
| --- | --- | --- | --- | --- | --- |
| L-PRF | 0.0098 |  |  |  |  |
| Age | 0.0000 | 0.0000 |  |  |  |
| Ulcer area | 0.0001 | 0.0000 | 0.0003 |  |  |
| Constant | -0.0142 | -0.0005 | -0.0012 | 0.0553 |  |
| Log($\sigma$) | 0.0000 | 0.0000 | 0.0000 | -0.0005 | 0.0052 |

#### Generalised gamma model

Appendix C Table 7 reports the results of the Generalised gamma accelerated failure time model for time to healing.

##### Appendix C Table 7. Generalised Gamma Accelerated Failure Time Model for Time to Healing in Patients with Plantar Ulcers

| **Variable** | **Coefficient** | **Standard error** | **95% confidence intervals** | | **Hazard ratio** |
| --- | --- | --- | --- | --- | --- |
| Constant | $\beta_{0}$= 1.3450 | 0.2320 | 0.8902 | 1.7998 | 3.8382 |
| Age | $\beta_{1}$= 0.0092 | 0.0031 | 0.0031 | 0.0153 | 1.0093 |
| Ulcer area | $\beta_{2}=$ 0.0462 | 0.0190 | 0.0089 | 0.0836 | 1.0473 |
| L-PRF | $\beta_{3}=$ -0.1500 | 0.0971 | -0.3403 | 0.0403 | 0.8607 |
| **Model statistics** |  |  |  |  |  |
| Scale parameter ($\sigma$) | $\sigma$ = 0.5111 | 0.05234 | 0.4181 | 0.6246 |  |
| Log($\sigma$) | -0.6712 | 0.1024 | -0.8720 | -0.4706 |  |
| Shape parameter ($\kappa$) | $\kappa$ = 0.3279 | 0.3801 | -0.4172 | 1.0729 |  |
| Log likelihood | -101.0490 |  |  |  |  |
| LR chi-squared | 18.31 |  |  |  |  |
| Prob > chi-squared | 0.0004 |  |  |  |  |
| Number of observations | 129 |  |  |  |  |

The time dependent transition probability for healing $tp(t)$ in each cycle draws upon the survivor function for each cycle $S(t)^{t}$.

$$tp(t)=1-S(t)^{t}/S(t)^{t-1}$$

$\kappa$ is the estimated shape parameter and $\sigma$ is the estimated scale parameter in the Generalised gamma accelerated failure time model for time to healing. The survivor function for each cycle $S(t)^{t}$ where $\kappa>0$ is:

$$S(t)^{t}= 1 - \Gamma(\kappa^{-2}(t/\lambda)^{\kappa/\sigma};\kappa^{-2})$$

where $\Gamma$ denotes the cumulative distribution function. For any cycle where $\kappa< 0$ the survivor function for that cycle is:

$$S(t)^{t}= \Gamma(\kappa^{-2}(t/\lambda)^{\kappa/\sigma};\kappa^{-2})$$

Lambda is calculated as follows:

$$\lambda=e^{{\beta_{0}}+\beta_{1}.Age+\beta_{2}.Ulcer area}$$

$$AFT=e^{\beta_{3}.LPRF}$$

$LPRF$ equals zero if the patient was treated with standard of care and one if the patient was treated with L-PRF.

Appendix C Table 8 presents the Cholesky decomposition matrix used to characterise the covariance structure of the parameter estimates in the Log-logistic accelerated time failure model.

##### Appendix C Table 8. Cholesky Decomposition Matrix for Covariance Structure in the Generalised Gamma Accelerated Time Failure Model

|  | L-PRF | Age | Ulcer area | Constant | Log($\gamma$) | $\kappa$ |
| --- | --- | --- | --- | --- | --- | --- |
| L-PRF | 0.0094 |  |  |  |  |  |
| Age | 0.0000 | 0.0000 |  |  |  |  |
| Ulcer area | 0.0000 | 0.0000 | 0.0004 |  |  |  |
| Constant | -0.0124 | -0.0004 | -0.0016 | 0.0538 |  |  |
| Log($\sigma$) | -0.0008 | 0.0000 | 0.0006 | -0.0051 | 0.0105 |  |
| $\kappa$ | 0.0040 | 0.0003 | -0.0028 | 0.0231 | -0.0269 | 0.1445 |

### Appendix D. Risk of recurrence

We calculate the relative risk of recurrence between L-PRF and normal saline dressing using data on the date of six-month follow up interview and questionnaire response on whether the trial ulcer had recurred on the same site. This gives a number of recurrences and time at risk of recurrence (i.e. the time between healing and the follow up appointment) for all patients who had healed according to the blinded assessor (n = 99). For those who had a recurred ulcer a random value was drawn. When recurrence occurred between healing and six-month follow up was not collected in the data, so we assume a uniform distribution, drawing a random value between 0 and the patients’ total time at risk. For each trial arm the weekly rate of recurrence is calculated as:

$$Weekly rate = recurred/total weeks at risk$$

The relative risk of recurrence is then calculated as:

$$RR = \frac{weekly rate^{L-PRF}}{weekly rate^{Saline}}$$

The standard error for the relative risk of recurrence is calculated as:

$$SE(RR)=\sqrt{\frac{1}{recurred^{Saline}}+\frac{1}{recurred^{L=PRF}}}$$

Appendix D Table 1. Relative risk of recurrence of trial ulcer

|  | Trial arm | |
| --- | --- | --- |
|  | L-PRF | SOC |
| Recurred | 14 | 19 |
| Did not recur | 34 | 32 |
| Total patients | 48 | 51 |
| Total weeks at risk | 869 | 873 |
| Weekly rate | 0.0161 | 0.0218 |
| Relative risk of recurrence | 0.7402 | |
| SE(RR) | 0.3522 | |

### Appendix E. Health state utilities

#### Literature review

A systematic review of the cost-effectiveness of intervention to prevent disability in leprosy found three studies (6). Two of these studies included quality of life measures; Ravi et al (2004) and Remme et al., 2021 (9,13), but neither was sufficient to inform health state utilities for our model.

Ravi et al. (2004) compared hospitalisation versus ambulatory care in the management of neuritis (involving steroids and a two-week period of rest) occurring in lepra reaction (i.e., acute inflammatory episodes that occur in individuals with leprosy that can worsen nerve damage) at a hospital in India (13). They used a used a 20-question survey derived from World Health Organization Quality of Life (WHOQOL), which had five domains (physical, psychological, level of independence, environment, and social). Each question had a maximum score of 5 except the question on pain in the physical domain which had a maximum of 6. Total maximum was 106, with a higher score indicating higher quality of life. Only 17 patients reported QoL, the mean pre-treatment scores in the ambulatory and inpatient group were 61.3 and 68.0, respectively. The mean post treatment scores in both groups were 77.7 and 78.8, respectively. While mapping the WHOQOL instrument to EQ-5D is conceptually possible, the instruments differ in design, purpose, and dimensions, scores for lepra reactions are not appropriate to inform the health state utilities of any of the states for our model.

Remme et al. (2021) undertook a modelling study across four tropical diseases, including leprosy (9). They use a WHO Disability Adjusted Life Year (DALY) weight of 0.152 for disabling leprosy. This encompasses leprosy patients with varying health complications requiring a wide range of treatments. For example, the study simulates cost effectiveness for interventions to cure leprosy, to treat reactions and ulcers, for those needing footwear and self-care education and for those needing reconstructive surgery. They apply a disability weight of 0 for leprosy patients without a disability. Similarly to the scores from Ravi et al (2004), the health state described in Remme et al (2021) is not similar enough to any of the states of our model to be used as disability weights from the Institute for Health Metrics and Evaluation (iHME) for the Global Burden of Disease study are for disfigurement due to leprosy rather than leprosy.

#### Ulcer and no ulcer

We obtain model health state utilities for the ulcer and no ulcer model states from data from the TABLE trial. Health-related quality of life (HRQoL) was measured using the EQ5D-3L instrument, which patients completed at randomisation, fortnightly until either the trial ulcer was deemed healed or trial period ended (whichever was first), and again at the six month follow up resulting in 600 EQ-5D observations.

Appendix E Table 1 presents baseline characteristics. Most patients are male, with a mean age of 54 and were diagnosed with leprosy 19 years ago on average. Half of patients have loss of motor function in either foot, and 90% have a deformity of the foot. One in five patients have more than one ulcer, which may contribute to worse HRQoL. For more than half of patients, the trial ulcer is recurrent, meaning they have had a previous ulcer on the same site as the existing trial ulcer. The mean time the trial ulcer has been unhealed is 50.75 weeks.

##### Appendix E Table 1: Baseline clinical and demographic characteristics: n, (%)

|  | Overall (n = 130) | Means for continuous variables  (sd) |
| --- | --- | --- |
| Female | 27 (20%) |  |
| Age*   - 18 - 39 - 40 - 59 - 60+ | 29 (22%)  49 (38%)  52 (40%) |  |
| Age |  | 54.03 (15.77) |
| Years since leprosy diagnosis |  | 19.73 (14.75) |
| Nerve enlarged in either leg | 76 (56%) |  |
| Loss motor function in either foot | 66 (51%) |  |
| Deformity in foot | 117 (90%) |  |
| More than one ulcer | 27 (21%) |  |
| Weeks the trial ulcer has been unhealed |  | 50.75 (88.67) |
| Trial ulcer is a recurrent ulcer | 81 (62%) |  |
| Baseline ulcer area cm2 |  |  |
| * age categories are derived from bsagecalc variable. The bsagecalc variable is continuous age variable. For patients where this variable is not known (n = 56), the best estimate is given | | |

Appendix E Table 2 shows mean EQ-5D recorded at each time point with measurements taken.

##### Appendix E Table 2. Mean EQ-5D (se) recorded at each time point with numbers of measurements taken

|  | 1 = Saline | 2 = L-PRF | Both |
| --- | --- | --- | --- |
| Baseline | 0.478, N = 65 | 0.504, N = 65 | 0.491, N = 130 |
| 5^th^ dressing change | 0.503, N = 64 | 0.573, N = 63 | 0.538, N = 127 |
| 9^th^ dressing change | 0.538, N = 52 | 0.564, N = 45 | 0.550, N = 97 |
| 13^th^ dressing change | 0.574, N = 36 | 0.489, N = 27 | 0.537, N = 63 |
| 17^th^ dressing change | 0.527, N = 19 | 0.521, N = 15 | 0.524, N = 34 |
| 21^st^ dressing change | 0.571, N = 16 | 0.408, N = 8 | 0.517, N = 24 |
| 6 month follow up | 0.603, N = 64 | 0.634, N = 61 | 0.618, N = 125 |

Disutility was modelled using a generalised estimating equation (GEE), as this accounts for distributional characteristics and repeated measures of the data. Model fit was assessed considering QIC measure of fit, QQ plots and the clinical/face validity of the results. The best fitting model was of Gaussian family, identity link, and assumes an exchangeable correlation structure. A variable for the ulcer being healed was also included, enabling us to estimate the utility for the ulcer and ulcer free state as:

$$g\left( {disutility}_{ij} \right)=\beta_{0}+\beta_{1}{Age}_{ij}+\beta_{2}{Female}_{ij}+\beta_{3}Ulcer healed$$

where $g$ is a specified link function (e.g., identity, log) which estimates disutility for individual $i$ at time point $j$. Age and sex were included as covariates as both were both statistically significant and were also the covariates used in the clinical trial analysis.

##### Appendix E Table 3: GEE GLM results with disutility as dependent variable, obs = 593

|  | **Estimate** | **Std. Error** | **Pr(>\|W\|)** |
| --- | --- | --- | --- |
| Intercept | 0.358947 | 0.050222 | 0.0000*** |
| Age | 0.001708 | 0.000842 | 0.0425* |
| Female (male = reference) | 0.107538 | 0.035596 | 0.0025** |
| Ulcer healed | -0.115445 | 0.020940 | 0.0000*** |
| Significance codes: 0 ‘***’ 0.001 ‘**’ 0.01 ‘*’ 0.05 ‘.’ 0.1 ‘ ’ 1  Family = gaussian  Link = identity  Correlation structure = exchangeable  Observations = 593  Number of clusters: 129 Maximum cluster size: 7 | | | |

The estimated mean utility (standard error) for the sample across all time points was 0.528 (0.549) for patients with an ulcer and 0.664 (0.010) with a healed ulcer.

#### Complicated ulcer and amputation

Ortegon et al. 2004 report mean utility values of 12 health states involving plantar ulcers and amputations arising from DFU from a time trade off study among members of the general public in the Netherlands (14). We apply these decrements to our baseline HRQoL from the trial to calculate utility values for the complicated ulcer and amputation states of the model. This assumes that these decrements are generalisable to the Nepalese population.

Ortegon et al (2004) do not define a complicated ulcer as such, so instead we apply the

##### Appendix E Table 4: Calculation of health state utilities using decrements from Ortegon et al (2004)

| This model health state | Ortegon et al (2004) health state | Decrement (compared to no ulcer) | This model health state utility calculation | This model health state utility (SE) |
| --- | --- | --- | --- | --- |
| No ulcer (reference value = 0.664) | No ulcer (reference value = 1) |  |  |  |
| Complicated ulcer | Active infected ulcer, 1+ toes amputated | 0.75 | 0.75 * 0.664 | 0.518 (0.054 |
| Amputation | One foot amputated | 0.79 | 0.79 * 0.664 | 0.546 (0.047) |
| Amputee | One foot amputated | 0.79 | 0.79 * 0.664 | 0.546 (0.047) |

### Appendix F. Costs and resource use

There is no cost associated with being healed, but patients incur one-off costs when they heal. These costs are reported in Appendix F Table 1, and are applied in the Markov trace when patients transition from the ulcer to the healed state.

##### Appendix F Table 1. Costs and resource use associated with ulcer healing

| Items | Price (2020 NRP) | Units/frequency |
| --- | --- | --- |
| Canvas shoes and microcellular rubber Insole | 1140 | One-off |
| Self-care assistance | 100 | One-off spread over two weeks |
|  |  |  |

In addition to the cost of treatment with Saline or L-PRF, patients with ulcers incur inpatient costs during their stay under treatment. These are detailed in Appendix F Table 2.

##### Appendix F Table 2. Costs and resource use for patients with ulcers

| Item | Cost (2020 NRP) | Units/frequency |
| --- | --- | --- |
| Bed Charge | 300 | Daily |
| Nursing Care | 250 | Daily |
| Food | 500 | Daily |
| Multivitamin | 4 | Per tablet, 2 tablets/daily |
| Vitamin C | 2 | Per tablet, 2 tablets/daily |
| Saline | 400 | Per procedure/twice weekly |
| L-PRF | 1800 | Per procedure/twice weekly |
| Antibiotics | 2.43 | Weekly |

Antibiotic costs are calculated as the average cost per week, using data from the trial (see Appendix F Table 3). Six patients required treatment for infected ulcers with antibiotics.

##### Appendix F Table 3. Antibiotic costs

| Antibiotics | Price (2020 NRP) | Cost per unit | Units/frequency | Total cost |
| --- | --- | --- | --- | --- |
| Injectable Ceftriaxone | 79/vial | 79 | 2 vials for 7 days | 1106 |
| Cotrimoxazole | 5/tablet | 5 | 2 tablets for 10 days | 100 |
| Ciprofloxacin | 7/tablet | 7 | 2 tablets for 15 days | 210 |
| Ofloxacin | 8/tablet | 8 | 2 tablets for 10 days | 160 |
| Doxycycline | 4.40/capsule | 4.4 | 2 capsule for 10 days | 88 |
| Ciprofloxacin | 7/tablet | 7 | 2 tablets for 10 days | 140 |

Patients with complicated ulcers incur the same costs as patients with simple (i.e., uncomplicated ulcers). Patients with complicated ulcers either require amputation or another surgery (which may be calcaneus paring, osteotomy, and sequestrectomy). The cost of amputation or other surgery are given in Appendix F Table 4 and are applied in the Markov model to the transition from the complicated state to amputation or to healed, respectively. Data to inform these costs are from the authors' institute in 2023.

There were four types of amputation surgeries provided at the authors' institute in 2023:

1 = Below knee amputation

2 = Forefoot amputation

3 = Multiple digit amputation

4 = Single digit amputation

A weight $W$ was calculated for each based on the proportion of amputation surgeries of each type that were provided. Weighted average amputation costs (WAAC) were calculated as:

$$WAAC=\frac{(C_{1}*W_{1})+(C_{2}*W_{2})+(C_{3}*W_{3})+(C_{4}*W_{4})}{W_{1}+W_{2}+W_{3}+W_{4}}$$

A cost $C$ for each was calculated and is based on the time required for the amputation (ranging from, on average, 20 to 60 minutes), the type of anathesia (spinal or ring block) and the cost of that type of anaesthesia. It also included surgery and recovery ward costs (i.e., meals, nursing and medical care in hospital, dressings and a daily bed charge) as well as medicine and investigation costs and post-surgery costs.

Three other types of surgeries were also provided in 2023 at the authors' institute after which ulcers were deemed healed. These are:

1= Calcaneus paring

2 = Osteotomy

3 = Sequestrectomy

The same method was used to calculated a weighted average costs for these non-amputation surgeries ($SC$):

$$SC=\frac{(C_{1}*W_{1})+(C_{2}*W_{2})+(C_{3}*W_{3})}{W_{1}+W_{2}+W_{3}}$$

A cost $C$ for each was calculated considering the time spent (30 to 45 minutes, on average) the type and cost of the anaesthesia (spinal or ankle block). As above, it also included surgery and recovery ward costs (i.e., meals, nursing and medical care in hospital, dressings and a daily bed charge) as well as medicine and investigation costs and post-surgery costs.

Costs were then deflated from 2023 NRP to 2020 NRP using Consumer Price Inflation data for Nepal from the World Bank (15).

Medication costs included pre-operative medications for both amputation and other surgeries. Other surgeries also had a course of ciprofloxacin.

##### Appendix F Table 4. Amputation and other surgery costs (2023 NRP)

| **Surgery** | **Surgery** | **Anaesthesia** | **Medication** |
| --- | --- | --- | --- |
| Below knee amputation (BKA) | 21000 | 5280 | 330 |
| Fore foot amputation | 15000 | 5280 | 330 |
| Amputation (Multiple digit) | 5000 | 330 | 330 |
| Amputation (Single digit) | 3000 | 330 | 330 |
| Calcanium Paring | 15000 | 5280 | 444.24 |
| Osteotomy Major | 15000 | 1320 | 444.24 |
| Sequestoctomy | 10000 | 1320 | 444.24 |

### Appendix G. Opportunity cost

1x and 3x GDP per capita have been widely applied as “cost-effectiveness thresholds” in the peer-reviewed literature to make assessments about the cost-effectiveness of healthcare interventions (16). In 2016, the World Health Organization distanced itself from recommendations to use these thresholds (17) as they do not reflect the opportunity cost of expenditure on health. Estimates of the opportunity cost of expenditure on health have started to become more widely used to make assessments about the cost-effectiveness of healthcare interventions (18). This enables decisionmakers to answer the question of whether funding the intervention would be expected to generate more health than other existing funded healthcare would generate with the same money.

It’s impossible to know exactly what is cut to free up funding for a new intervention, and so instead the opportunity cost of expenditure can be informed by econometric estimates of the marginal productivity the healthcare system. Such estimates are available for a growing number of countries (19), but the data requirements for estimating these values is high and no such study has been undertaken for Nepal. Estimates for Nepal for 2015 are available from a study that uses international data (20). We use an estimate from Lomas et al (2022), who project these values based on Ochalek et al (2018) (21). We use an estimate of k in Nepal in 2020 in 2017 USD, and this is converted to 2017 NRP using the World Bank exchange rate, then inflated to 2021 NRP using Consumer Price Inflation in Nepal also from the World Bank.

Lomas et al (2022) estimate that for every 38,970 NRP spent on healthcare, one disability adjusted life year (DALY) is averted (21). Therefore, in order for L-PRF to generate a net health benefit, it must avert one DALY (or, equivalently, gain one QALY) at a cost lower than 38,970 NRP.

### Appendix H. Results of L-PRF using the other three models

##### Appendix H Table 1. Incremental net health benefit by model (probabilistic sensitivity analysis)

|  | Weibull | Lognormal | Loglogistic | Generalised gamma |
| --- | --- | --- | --- | --- |
| Incremental net health benefit per patient | - 0.167 | - 0.360 | - 0.321 | - 0.345 |
| Population incremental net health benefit | -166.568 | - 1,158.017 | - 1,032.764 | - 1,110.229 |

##### Appendix H Table 2. Total costs and effects of treatment (Weibull probabilistic sensitivity analysis)

| **2020 Nepalese Rupees** | | | | | | |
| --- | --- | --- | --- | --- | --- | --- |
|  | Cost | 95% CIs | | QALYs | 95% CIs | |
| Standard | 125,092 | 102,040 | 151,924 | 4.013 | 3.766 | 4.239 |
| L-PRF | 131,824 | 69,038 | 194,448 | 4.019 | 3.772 | 4.253 |
| Difference | 6,731 | -58,627 | 63,563 | 0.006 | - 0.004 | 0.019 |
| ICER | 1,093,373 | | | | | |
| **Incremental net health benefit at a threshold of 38,970 (2020 Nepalese rupees)** | | | | | | |
| Incremental net health benefit per patient | | | | - 0.167 (-1.64, 1.52) | | |
| Population incremental net health benefit | | | | -166.568 (163,643.23, 151,929.84) | | |

*95% confidence intervals were calculated from the Monte Carlo simulation.

##### Appendix H Table 3. Total costs and effects of treatment (Lognormal probabilistic sensitivity analysis)

| **2020 Nepalese Rupees** | | | | | | |
| --- | --- | --- | --- | --- | --- | --- |
|  | Cost | 95% CIs | | QALYs | 95% CIs | |
| Standard | 124,564 | 101,727 | 151,400 | 4.017 | 3.781 | 4.266 |
| L-PRF | 138,846 | 79,309 | 204,896 | 4.024 | 3.783 | 4.271 |
| Difference | 14,282 | - 51,300 | 78,773 | 0.006 | - 0.005 | 0.019 |
| ICER | 2,212,159.57 | | | | | |
| **Incremental net health benefit at a threshold of 38,970 (2020 Nepalese rupees)** | | | | | | |
| Incremental net health benefit per patient | | | | - 0.360 (-2.03, 1.33) | | |
| Population incremental net health benefit | | | | - 1,158.017 (-202,548.50, 133,426.91) | | |

*95% confidence intervals were calculated from the Monte Carlo simulation.

##### Appendix H Table 4. Total costs and effects of treatment (Loglogistic probabilistic sensitivity analysis)

| **2020 Nepalese Rupees** | | | | | | |
| --- | --- | --- | --- | --- | --- | --- |
|  | Cost | **95% CIs** | | **QALYs** | **95% CIs** | |
| Standard | 124,753 | 100,664 | 150,799 | 4.020 | 3.778 | 4.270 |
| L-PRF | 137,527 | 77,198 | 201,365 | 4.027 | 3.786 | 4.280 |
| Difference | 12,774 | - 23,466 | 50,566 | 0.007 | 0.008 | 0.010 |
| ICER | 1,909,140.07 | | | | | |
| **Incremental net health benefit at a threshold of 38,970 (2020 Nepalese rupees)** | | | | | | |
| Incremental net health benefit per patient | | | | - 0.321 (-1.85, 1.25) | | |
| Population incremental net health benefit | | | | - 1,032.764 (-185,257.59, 125,343.86) | | |

*95% confidence intervals were calculated from the Monte Carlo simulation.

##### Appendix H Table 5. Total costs and effects of treatment (Generalised gamma probabilistic sensitivity analysis)

| **2020 Nepalese Rupees** | | | | | | |
| --- | --- | --- | --- | --- | --- | --- |
|  | Cost | 95% CIs | | QALYs | 95% CIs | |
| Standard | 125,041 | 102,405 | 151,902 | 4.020 | 3.787 | 4.246 |
| L-PRF | 138,749 | 73,755 | 204,997 | 4.027 | 3.793 | 4.259 |
| Difference | 13,708 | - 54,550 | 74,873 | 0.007 | - 0.004 | 0.021 |
| ICER | 2,084,749.15 | | | | | |
| **Incremental net health benefit at a threshold of 38,970 (2020 Nepalese rupees)** | | | | | | |
| Incremental net health benefit per patient | | | | - 0.345 (-1.92, 1.42) | | |
| Population incremental net health benefit | | | | - 1,110.229 (-192,427.84, 141,530.51) | | |

*95% confidence intervals were calculated from the Monte Carlo simulation.

### References

1. Drummond M, Sculpher M, Claxton K, Stoddart G, Torrance G. Methods for the Economic Evaluation of Health Care Programmes [Internet]. 4th ed. Oxford: Oxford Medical Publications; 2015 [cited 2015 Nov 27]. Available from: http://www.amazon.co.uk/Methods-Economic-Evaluation-Health-Programmes/dp/0199665885

2. Philips Z, Ginnelly L, Sculpher M, Claxton K, Golder S, Riemsma R, et al. Review of guidelines for good practice in decision-analytic modelling in health technology assessment. Health Technol Assess [Internet]. 2004 [cited 2024 Nov 22];8(36). Available from: https://pubmed.ncbi.nlm.nih.gov/15361314/

3. Wu B, Wan X, Ma J. Cost-effectiveness of prevention and management of diabetic foot ulcer and amputation in a health resource-limited setting. J Diabetes [Internet]. 2018 Apr 1 [cited 2025 Jan 22];10(4):320–7. Available from: https://onlinelibrary.wiley.com/doi/full/10.1111/1753-0407.12612

4. Romero Prada M, Roa C, Alfonso P, Acero G, Huérfano L, Vivas-Consuelo D. Cost-effectiveness analysis of the human recombinant epidermal growth factor in the management of patients with diabetic foot ulcers. Diabet Foot Ankle [Internet]. 2018 Jan 1 [cited 2025 Jan 22];9(1). Available from: https://www.tandfonline.com/doi/abs/10.1080/2000625X.2018.1480249

5. Cárdenas MK, Mirelman AJ, Galvin CJ, Lazo-Porras M, Pinto M, Miranda JJ, et al. The cost of illness attributable to diabetic foot and cost-effectiveness of secondary prevention in Peru. BMC Health Serv Res [Internet]. 2015 Oct 26 [cited 2025 Jan 22];15(1):1–10. Available from: https://link.springer.com/articles/10.1186/s12913-015-1141-4

6. van Veen NHJ, McNamee P, Richardus JH, Smith WCS. Cost-Effectiveness of Interventions to Prevent Disability in Leprosy: A Systematic Review. PLoS One [Internet]. 2009 Feb 20 [cited 2024 Dec 11];4(2):e4548. Available from: https://journals.plos.org/plosone/article?id=10.1371/journal.pone.0004548

7. Ravi HN, George R, Eapen EP, Pulimood SA, Gnanamuthu C, Jacob M, et al. A comparison of economic aspects of hospitalization versus ambulatory care in the management of neuritis occurring in lepra reaction. International Journal of Leprosy and Other Mycobacterial Diseases [Internet]. 2004 Dec [cited 2025 Jul 15];72(4):448–56. Available from: https://pubmed.ncbi.nlm.nih.gov/15755199/

8. Seboka G, Saunderson P. Cost-effective footwear for leprosy control programmes: A study in rural Ethiopia. Lepr Rev [Internet]. 1996 [cited 2025 Jul 15];67(3):208–16. Available from: https://pubmed.ncbi.nlm.nih.gov/8885615/

9. Remme JH, Feenstra P, Level P, Medici AC, Morel CM, Noma M, et al. Tropical diseases targeted for elimination: Chagas disease, lymphatic filariasis, onchocerciasis, and leprosy. International Journal of Biomedical and Health Sciences. 2021;(9).

10. Collazo Herrera M, Sosa Lorenzo I, González LF, García Díaz D, Miranda BC. Economic evaluation of Heberprot P® treatment for diabetic foot ulcers at Havana Diabetes Care Center, 2012-2014. Pharmacoeconomics - Spanish Research Articles [Internet]. 2017 Jul 1 [cited 2025 Jul 15];14(2):59–66. Available from: https://link.springer.com/article/10.1007/s40277-017-0071-4

11. Govindasamy K, Darlong J, Watson SI, Gill P. Prevalence of plantar ulcer and its risk factors in leprosy: a systematic review and meta-analysis. J Foot Ankle Res [Internet]. 2023 Dec 1 [cited 2024 Sep 20];16(1):77. Available from: /pmc/articles/PMC10641946/

12. Napit IB, Shrestha D, Choudhury S, Gkini E, Ilozumba O, Gill P, et al. A randomised Trial of Autologous Blood products, leukocyte and platelet-rich fibrin (L-PRF), to promote ulcer healing in LEprosy: The TABLE trial. PLoS Negl Trop Dis [Internet]. 2024 May 1 [cited 2024 May 30];18(5):e0012088. Available from: https://journals.plos.org/plosntds/article?id=10.1371/journal.pntd.0012088

13. Ravi HN, George R, Eapen EP, Pulimood SA, Gnanamuthu C, Mary Jacob, et al. A Comparison of Economic Aspects of Hospitalization Versus Ambulatory Care in the Management of Neuritis Occurring in Lepra Reaction. Int J Lepr. 2004;72(4):448–56.

14. Ortegon MM, Redekop WK, Niessen LW. Cost-Effectiveness of Prevention and Treatment of the Diabetic FootA Markov analysis. Diabetes Care [Internet]. 2004 Apr 1 [cited 2024 Nov 25];27(4):901–7. Available from: https://dx.doi.org/10.2337/diacare.27.4.901

15. Inflation, consumer prices (annual %) - Nepal | Data [Internet]. [cited 2024 Nov 25]. Available from: https://data.worldbank.org/indicator/FP.CPI.TOTL.ZG?end=2023&locations=NP&start=2021

16. Leech AA, Kim D, Cohen J, Neumann PJ. Use and Misuse of Cost-Effectiveness Analysis Thresholds in Low- and Middle-Income Countries: Trends in Cost-per-DALY Studies. Value in Health [Internet]. 2018; Available from: https://www.sciencedirect.com/science/article/pii/S1098301518300160

17. Bertram MY, Lauer JA, De Joncheere K, Edejer T, Hutubessy R, Kieny MP, et al. Cost-effectiveness thresholds: pros and cons. Bull World Health Organ. 2016 Dec;94(12):925–30.

18. Horton S, Gelband H, Jamison D, Levin C, Nugent R, Watkins D. Ranking 93 health interventions for low- and middle-income countries by cost-effectiveness. Smith Fawzi MC, editor. PLoS One [Internet]. 2017 Aug 10 [cited 2018 Feb 15];12(8):e0182951. Available from: http://dx.plos.org/10.1371/journal.pone.0182951

19. Edney LC, Lomas J, Karnon J, Vallejo-Torres L, Stadhouders N, Siverskog J, et al. Empirical Estimates of the Marginal Cost of Health Produced by a Healthcare System: Methodological Considerations from Country-Level Estimates. Pharmacoeconomics [Internet]. 2022 Jan 1 [cited 2022 Oct 13];40(1):31–43. Available from: https://pubmed.ncbi.nlm.nih.gov/34585359/

20. Ochalek J, Lomas J, Claxton K. Estimating health opportunity costs in low-income and middle-income countries: a novel approach and evidence from cross-country data. BMJ Glob Health. 2018 Nov;3(6):e000964.

21. Lomas J, Claxton K, Ochalek J. Accounting for country- and time-specific values in the economic evaluation of health-related projects relevant to low- and middle-income countries. Health Policy Plan [Internet]. 2022 Jan 13 [cited 2022 Feb 21];37(1):45–54. Available from: https://doi.org/10.1093/heapol/czab104

1. Grade 0 – absence of disability and no visible damage or deformities on eyes, hands and feet; Grade 1 – loss of protective sensibility in the eyes, hands or feet, but no visible damage or deformities; Grade 2 – presence of deformities or visible damage to the eyes, visible damage on hands or feet (hand with ulcerations and/or traumatic, resorption, claw, fallen hand, ulcers; feet with trophic and/or traumatic injuries, resorption, claw, foot drop, ulcers, ankle contracture). [↑](#footnote-ref-1)
